# Hyperosmotic stress-induced microtubule disassembly in *Chlamydomonas reinhardtii*

**DOI:** 10.1186/s12870-022-03439-6

**Published:** 2022-01-22

**Authors:** Lee Mei Ng, Shinichiro Komaki, Hideyuki Takahashi, Takashi Yamano, Hideya Fukuzawa, Takashi Hashimoto

**Affiliations:** 1 Graduate School of Science and Technology, Nara Institute of Science and Technology, Ikoma, Nara, 630-0192 Japan; 2 Graduate School of Biostudies, Kyoto University, Kyoto, Kyoto 606-8502 Japan

## Abstract

**Background:**

Land plants respond to drought and salinity by employing multitude of sophisticated mechanisms with physiological and developmental consequences. Abscisic acid-mediated signaling pathways have evolved as land plant ancestors explored their habitats toward terrestrial dry area, and now play major roles in hyperosmotic stress responses in flowering plants. Green algae living in fresh water habitat do not possess abscisic acid signaling pathways but need to cope with increasing salt concentrations or high osmolarity when challenged with adverse aquatic environment. Hyperosmotic stress responses in green algae are largely unexplored.

**Results:**

In this study, we characterized hyperosmotic stress-induced cytoskeletal responses in *Chlamydomonas reinhardtii*, a fresh water green algae. The *Chlamydomonas* PROPYZAMIDE-HYPERSENSITEVE 1 (PHS1) tubulin kinase quickly and transiently phosphorylated a large proportion of cellular α-tubulin at Thr349 in G1 phase and during mitosis, which resulted in transient disassembly of microtubules, when challenged with > 0.2 M sorbitol or > 0.1 M NaCl. By using *phs1* loss-of-function algal mutant cells, we demonstrated that transient microtubule destabilization by sorbitol did not affect cell growth in G1 phase but delayed mitotic cell cycle progression. Genome sequence analyses indicate that *PHS1* genes evolved in ancestors of the Chlorophyta. Interestingly, *PHS1* genes are present in all sequenced genomes of freshwater Chlorophyta green algae (including *Chlamydomonas*) but are absent in some marine algae of this phylum.

**Conclusion:**

PHS1-mediated tubulin phosphorylation was found to be partly responsible for the efficient stress-responsive mitotic delay in *Chlamydomonas* cells. Ancient hyperosmotic stress-triggered cytoskeletal remodeling responses thus emerged when the *PHS1* tubulin kinase gene evolved in freshwater green algae.

**Supplementary Information:**

The online version contains supplementary material available at 10.1186/s12870-022-03439-6.

## Background

Colonization of terrestrial environment during land plant evolution required innovations to adapt to desiccation, fluctuation temperatures, and high UV irradiation. For example, key genes that increase resistance to desiccation, such as abscisic acid (ABA) receptor genes, were probably gained by horizontal gene transfer from soil bacteria in the earliest-diverging embryophyte ancestors [1]. Diverse green algae dwell in terrestrial freshwater environments. Although they do not possess effective stress-response adaptation mechanisms of land plants, freshwater green algae need to respond and adapt to occasional dehydration and salt challenges. Such primitive or ancient stress responsive mechanisms are largely unknown.

Propyzamide hypersensitive 1 (PHS1) was discovered in a flowering plant *Arabidopsis thaliana* that mediates hyperosmotic stress to remodel the microtubule cytoskeleton [2]. PHS1 consists of an N-terminal tubulin kinase domain and a C-terminal phosphatase domain [3]. Under unstressed growth conditions, the phosphatase activity suppresses the tubulin kinase activity, thereby maintaining the kinase in an inactive state. Upon moderate hyperosmotic stress, the tubulin kinase is immediately activated and phosphorylates Thr349 of α-tubulin. Since the phosphorylated tubulin does not readily assemble into a microtubule polymer, cellular microtubules containing phosphorylated tubulins become shortly disassembled [3]. In vitro, purified tubulin populations containing the Thr349-phosphorylated forms do not readily polymerize [4]. However, it is not known how such microtubule disassembly affects plant performance under stress, and whether PHS1-mediated stress responses occur in other plants, especially in aquatic non-land plants. In this study, we characterized a PHS1 tubulin kinase in *Chlamydomonas reinhardtii,* a model freshwater algal species belonging to the Chlorophytes phylum.

## Results

### Establishment of synchronized *Chlamydomonas* cell cultures

**Fig. 1:**
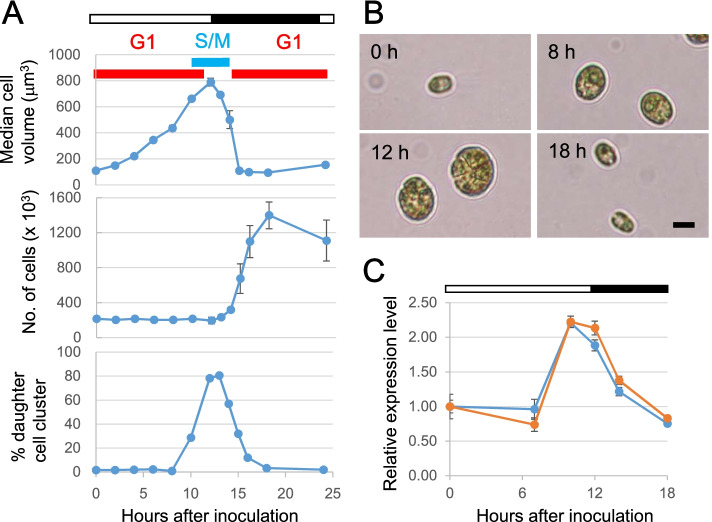
Synchronized *Chlamydomonas* cell cultures. (**a**) Cell growth and formation of daughter cells. Estimated cell cycle phases are indicated. Cells were illuminated (white bar) or grown in the dark (black bar). (**b**) Cell morphology at the indicated time point after inoculation, analyzed by light microscopy. Bar: 10 μm. (**c**) Relative expression levels of *CDKB* (blue) and *CYCB* (orange) during culture. White and black bars respective indicate light and dark periods. Error bars indicate standard deviations from three biological replicates

In order to analyze cell growth and cell biological phenotypes separately in G1 phase and mitosis, growth of *Chlamydomonas reinhardtii* (CC-4533 strain) was synchronized by adjusting an inoculum at the beginning of the culture and by alternating the illumination in a 12 h light-12 h dark cycle. The number, the size and the morphology were analyzed by using a coulter counter and a light microscopy. The cell size showed Gaussian distribution patterns (Fig. S1), and the size distribution peaks increased while maintaining the initial cell number until the 12th h, when the cells increased the cell volume approximately eight-fold (Fig. 1A). After the onset of the dark period at the 12th h, the number of free daughter cells began to increase and, by the 16th h, the cell number increase stopped, and all the cells were small as the beginning of the culture. Microscopic images of individual cells at each cell cycle stage supported the coulter counter analyses, and further showed that *Chlamydomonas* cells formed daughter cell clusters that peaked at the 12th to 13th h (Fig. 1B). Individual daughter cells in a cell cluster were released (i.e., hatched) after the 14th h. The cell number analysis by the coulter counter does not differentiate cell aggregates from free single cells. Expression patterns of two cell-cycle regulated genes were analyzed by reverse transcription qPCR. *CDKB* and *CYCB* are a cyclin-dependent kinase gene and a cyclin gene, respectively, which are specifically up-regulated during the S/M phase of the *Chlamydomonas* cell cycle [5, 6]. The expression levels of these two genes were low at the beginning of the synchronized culture, increased sharply at the 10th h before the start of the dark period, and then started to decrease after 12th h (Fig. 1C). Altogether, these analyses show that a high level of cell cycle synchronization was attained and that under our synchronization conditions the cells are in the G1 phase from the start of the culture to the 10th h, while the mitosis occurs between the 10th and the 13th h.

### Tubulin is phosphorylated under salt and osmotic stress in *Chlamydomonas*

**Fig. 2:**
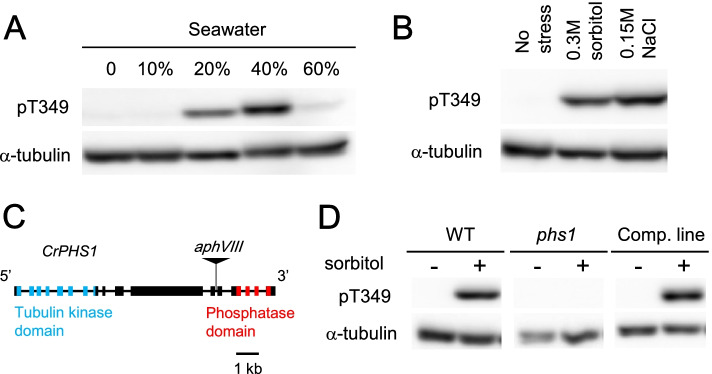
PHS1 phosphorylates α-tubulin in response to hyperosmotic stress. Immunoblots using an antibody specifically recognizing α-tubulin phosphorylated at Thr349 (pT349) or a general antibody for α-tubulin. Experiments were repeated at least once with similar results. (**a**) Cells were treated with sea water at the indicated levels for 10 min. (**b**) Cells were treated with 0.3 M sorbitol or 0.15 M NaCl for 10 min. (**c**) Gene structure of *C. reinhardtii*, *PHS1*. The boxes and connecting lines indicate exons and introns, respectively. Insertion site of the *aphVIII* gene is shown. The tubulin kinase and phosphatase domains are shown in blue and red boxes, respectively. (**d**) Sorbitol treatment at 0.2 M for 10 min triggered tubulin phosphorylation in wild-type and complemented cells, but not in *phs1* mutant cells

Commercial artificial seawater was applied to the *Chlamydomonas* cells in G1 phase for 10 min at the concentrations of 10, 20, 40 and 60%. Tubulin phosphorylation was analyzed by a specific phosphor-tubulin antibody. This antibody was raised against a phospho-peptide (N-FVDWCPpTGFKCGIN-C) targeting the Thr349 of *Arabidopsis* α-tubulin [4]. This peptide sequence is perfectly conserved in the two α-tubulins of *C. reinhardtii*. The immunoblot detected α-tubulin phosphorylation at Thr349 at 20 and 40% of seawater but not at 0, 10 and 60% (Fig. 2A). Phosphorylation of α-tubulin at Thr349 was previously detected in the non-targeted phosphor-proteome analysis of *C. reinhardtii* proteins [7]. The salt concentration at 40% seawater corresponds to ~ 0.17 M NaCl equivalent. At 60% seawater, the cells did not grow at all, indicating that the treated cells are physiologically dormant or dying.

Both sorbitol and salt induce hyperosmotic stress to the cells, while the effects of high concentrations of salt also involve cellular ion imbalance [8]. When the effects of sorbitol and NaCl were compared at the similar osmolarity, comparable levels of the tubulin phosphorylation were observed. When *Chlamydomonas* cells were treated by 0.3 M sorbitol or 0.15 M NaCl, tubulins were phosphorylation at similar levels after 10 min (Fig. 2B). These concentrations of sorbitol and NaCl, however, affected the cell growth differently; NaCl at 0.15 M inhibited the cell volume increase much more severely than sorbitol at 0.3 M (Fig. S2). These results suggest that the tubulin phosphorylation is mainly caused by hyperosmotic stress and that the inhibition of cell growth by salt involves effects other than the osmotic stress.

Since the CC-4533 strain possesses a *cw15* wall-deficient mutation [9], other laboratory strains of *C. reinhardtii* were also examined. The C9 and CC-125 strains possess normal cell wall, whereas the CC-5155, CC-5325 and CC-4533 strains also are defective in cell wall components (Chlamydomonas Resource Center; https://www.chlamycollection.org/). These strains were treated either with NaCl or sorbitol for 20 min, and then analyzed for tubulin phosphorylation. The immunoblot results in Fig. S3 showed that all strains, regardless of intact or defective cell walls, phosphorylated α-tubulin upon treatment with 0.1–0.2 M NaCl and with 0.2 M sorbitol. Thus, the stress-induced tubulin phosphorylation does not depend on the cell wall status.

### PHS1 mediates stress-induced tubulin phosphorylation

The single-copy *PHS1* gene (Cre08.g375000) is located at chromosome 8 of *C. reinhardtii*, consists of 17 exons and 16 introns, and potentially encodes a polypeptide of 1905 amino acid residues. The positions of introns were supported by an RNAseq analysis (H. Fukuzawa, personal communication). To examine whether *Chlamydomonas* PHS1 is responsible for the observed tubulin phosphorylation, as shown in Arabidopsis [3], a *PHS1* insertion mutant, in which paromomycin resistance *aphVIII* gene was inserted in the *PHS1* locus, was obtained (Fig. 2C). A *PHS1* genomic fragment was transformed to this *phs1–1* allele, hereafter simply described as *phs1*. Genomic PCR analyses confirmed that *PHS1* was disrupted by *aphVIII* gene in *phs1* and its complementation line, whereas an intact *PHS1* region of 1.2 kb was present in the genomes of both wild type and the complementation line (Fig. S4). Treatment of 0.2 M sorbitol for 10 min induced tubulin phosphorylation in wild type and the complementation line, but not in *phs1*, indicating recovery of a tubulin kinase activity by the introduction of the wild-type *PHS1* copy in the *PHS1* null mutant (Fig. 2D). Thus, PHS1 is solely responsible for the hyperosmotic stress-induced α-tubulin phosphorylation at Thr349 in *Chlamydomonas* cells.

### Sorbitol-triggered tubulin phosphorylation is rapid and transient, and occurs in G1 phase and mitosis

**Fig. 3:**
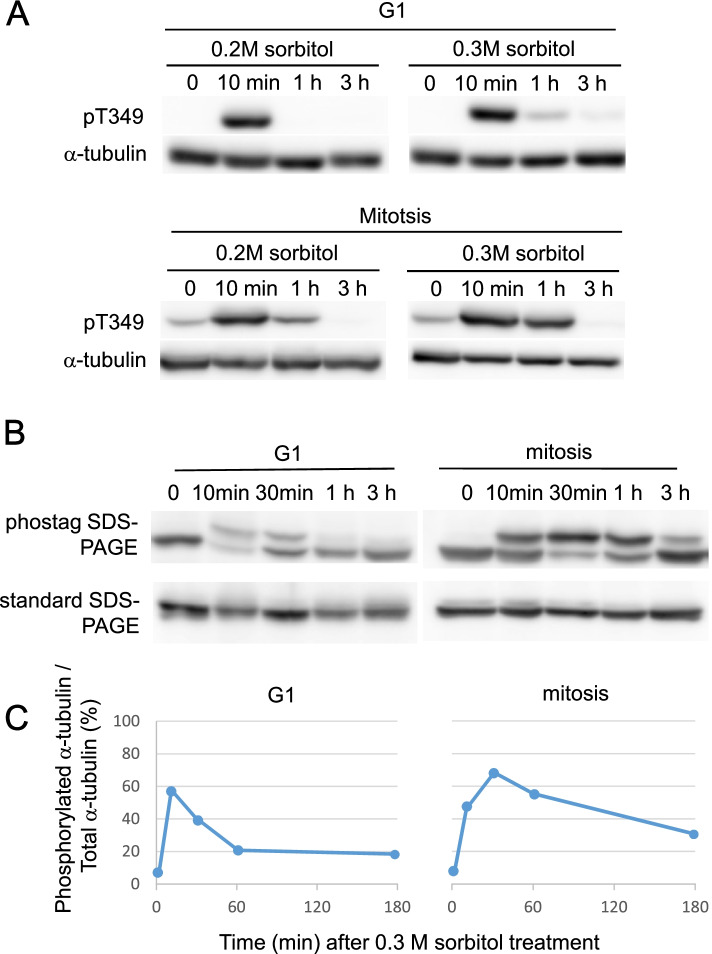
Time courses of stress-induced tubulin phosphorylation. Experiments were repeated at least once with similar results. (**a**) Cells were treated by 0.2 M or 0.3 M sorbitol at G1 or mitosis. Immunoblots used antibodies indicated in the legend of Fig. 2. (**b**) Phos-tag SDS-PAGE blots show that phosphorylated tubulins (upper bands) constituted large tubulin fractions after the 0.3 M sorbitol stress. Standard SDS-PAGE blots, which did not distinguish tubulin modifications, are also shown for comparison. General anti-α-tubulin antibody was used for analyses. (**c**) Percentages of phosphorylated tubulins in total tubulins were quantified by image analyses of (**b**)

Effects of sorbitol were studied in G1 phase (at the 6th h after inoculation) and mitosis (at the 11th h; Fig. 3A). In the G1 phase cells, sorbitol at 0.2 M and 0.3 M induced high tubulin phosphorylation after 10 min of stress treatment; thereafter, tubulins were swiftly dephosphorylated within 1 h. In the mitotic cells, similar patterns of tubulin phosphorylation and dephosphorylation were observed, but there were low levels of phosphorylated tubulins often detected even in the absence of the stress, and tubulin dephosphorylation took somewhat longer time to complete.

To determine how much tubulins are phosphorylated, we used a phosphor-protein-interacting chemical, the phos-tag [10], to separate phosphorylated and non-phosphorylated proteins in the phos-tag-containing SDS PAGE gels (Fig. 3B, C). The peaks of tubulin phosphorylation occurred after 10 min (G1 phase) and 30 min (mitosis) of 0.3 M sorbitol treatment, and more than 60% of cellular tubulins were phosphorylated at the peaks. Lower levels of tubulin phosphorylation in the G1 phase cells may reflect that the flagellar microtubules are highly stable and only exchange with cellular tubulins at the flagellar tips [11, 12].

These results demonstrate that hyperosmotic stress induces rapid, massive, and transient tubulin phosphorylation in both G1 phase and mitotic cells of *C. reinhardtii*.

### Microtubules are transiently disassembled by PHS1 in sorbitol-treated cells

To examine the consequences of stress-induced transient tubulin phosphorylation on microtubule organization, cytoplasmic microtubules were labeled by both anti-α-tubulin antibody and anti-acetylated-α-tubulin antibody. Acetylated tubulins are preferentially present in stable and long-lived microtubules and protect microtubules from mechanical breakage [13]. In G1 phase cells, four microtubule bundles, called “rootlets” extend from the basal body toward the other end of the cell body along the cell periphery, and are known to contain acetylated tubulins, indicating their partial stability [14].

**Fig. 4:**
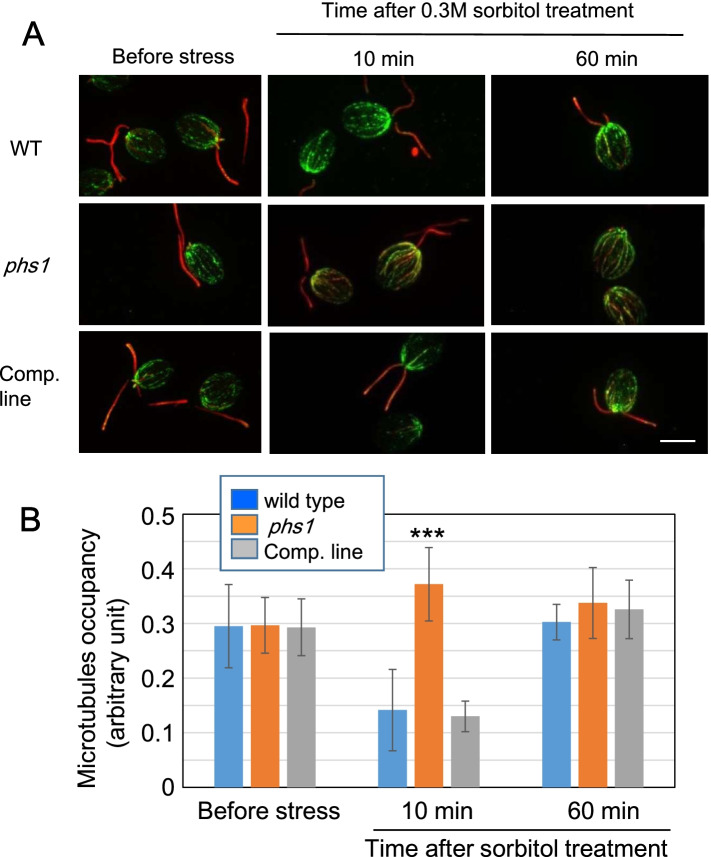
Hyperosmotic stress transiently depolymerizes interphase microtubules in a PHS1-dependent manner. (**a**) Immunohistochemical images of microtubules (green) and acetylated microtubules (red), which mainly represented flagella microtubules, after application of 0.3 M sorbitol in wild type (WT), *phs1* mutant, and a complimented line (comp. line). Bar: 10 μm. (**b**) Quantification of microtubule polymer amounts. ***; statistically significant difference at *p* < 0.01 by one-way ANOVA with Tukey-HSD post-hoc test, when compared with wild-type values. Error bars indicate standard deviations from ten cells for each genotype

In the non-stressed control cells, a few thick microtubule bundles that often contain some acetylated tubulins were discernible and most likely represent microtubule rootlets (Fig. 4A). Flagellar microtubules are heavily acetylated [15], and were also strongly stained in this study. Microtubule organizations were indistinguishable among wild type, *phs1*, and its complementation line. Upon sorbitol treatment for 10 min, microtubules in wild type and the complementation line were considerably depolymerized, whereas such rapid microtubule disassembly did not occur in *phs1*. After 1 h of the stress treatment, microtubule organization in wild type and the complementation line recovered to the initial non-stress levels. These results indicate that the PHS1-mediated tubulin phosphorylation leads to transient microtubule instability in G1 phase cells. Such stress-trigged microtubule reorganization is effective only for a short time window of less than 1 h. Microtubule density in each cells was calculated as “microtubule occupancy”, which was derived by dividing the microtubule area by whole cell area, after conversion of original α-tubulin immuno-stained images to binary images by Image J (Fig. S5). Quantification and statistical analysis of the microtubule polymer density in cells confirmed these results (Fig. 4B).

### Cell division, but not cell growth, is affected by PHS1-mediated microtubule destabilization

**Fig. 5:**
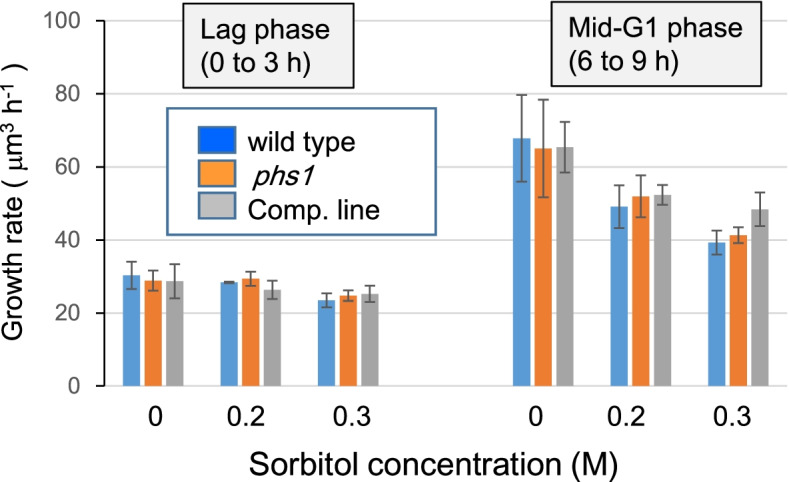
Sorbitol stress does not inhibit cell growth during G1. Growth rates in terms of increase in cell volume were monitored during a lag phase and a mid-G1 phase when cells of three genotypes (wild type, *phs1* mutant, a complementation line) were cultured in the presence of 0 M, 0.2 M, or 0.3 M sorbitol. Error bars indicate standard deviations from three biological replicates

Since the above results show that hyperosmotic stress depolymerizes cytoplasmic microtubules for a short period (less than 1 h), we first examined whether such a transient microtubule destabilization affects cell growth during G1 phase. In *Chlamydomonas* cells in which cell growth and cell division are partially uncoupled, how fast cells grow during a prolonged G1 phase determines how many fission cycles occur after the light period [16]. Hyperosmotic stress was applied at the onset of synchronization culture by inoculating *Chlamydomonas* cells into culture media containing 0.2 M or 0.3 M sorbitol. During the G1 phase duration up to 9 h, no increase in the cell number in culture was observed while the individual cell size kept increasing, as monitored by the coulter counter. Growth rates (i.e. increase in cell volume) were calculated for the initial lag phase (up to 3 h after inoculation) and for the later accelerated growth phase (from 6 to 9 h). Increasing concentrations of sorbitol inhibited cell growth more strongly during the accelerated growth phase, but the growth rates for both phases were indistinguishable among wild type, *phs1*, and the complementation line (Fig. 5). These results show that PHS1 does not play any obvious roles in cell growth during G1 phase of sorbitol-stressed cells.

**Fig. 6:**
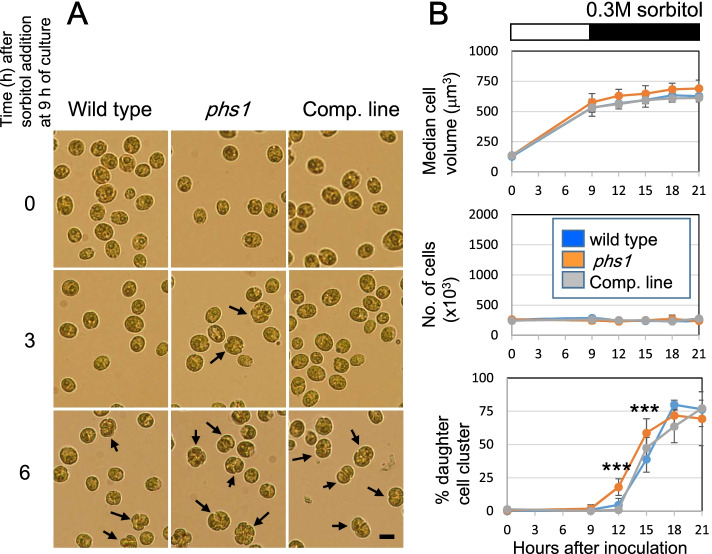
Hyperosmotic stress-triggered delay in mitotic progression is partly mediated by PHS1. (**a**) Microscopic cell images of three genotypes (wild type, *phs1* mutant, a complementation line) after 0.3 M sorbitol treatment at 9 h of culture. Arrows indicate clusters of newly divided daughter cells. (**b**) Cell growth and formation of daughter cell clusters. Sorbitol at 0.3 M was applied at 9 h after the start of culture (during the period indicated by a black bar). ***; statistically significant difference at *p* < 0.01 by one-way ANOVA with Tukey-HSD post-hoc test, when compared with wild-type values. Error bars indicate standard deviations from five biological replicates

Next, dividing cells were targeted for osmotic stress, by applying 0.3 M sorbitol at the 9th h, just before the onset of mitosis. Under non-stressed conditions, *Chlamydomonas* cells transiently formed cell clusters containing four or eight daughter cells encapsulated within the mother cell wall, from which free daughter cells hatched within a few hours (see Fig. 1a). Bright field microscopic images showed that sorbitol stress treatment strongly inhibited hatching in three genotypes, so that newly divided daughter cells remained associated for several hours (Fig. 6A). In the time frame of our experiments (up to 12 h in the stressed conditions), no free daughter cells were released, as indicated by no net increase in the combined number of single cells and cell aggregates after the stress (Fig. 6B). Formation of daughter cell clusters were delayed, but not strongly inhibited, by the stress in three genotypes, indicating that mild hyperosmotic stress delayed progression of mitosis. However, we found that mitotic progression was less affected in the *phs1* cells, compared to wild-type cells and the complemented cells. Formation of daughter cells were already observed after 3 h of stress application (the 12th h after the onset of culture) in the *phs1* cells, but not in wild-type cells and the complimented cells (Fig. 6A, B). These results indicate that the delayed cell cycle progression after the stress was partly caused by the PHS1 functions.

### *PHS1* genes in algae

Presence of a functional *PHS1* gene in *C. reinhardtii* prompted us to search possible presence of algal *PHS1* genes in the sequenced genomes. Since the tubulin kinase domain possessing an atypical kinase domain with high homology to the unique actin-fragmin kinase domain [17] is a hallmark of the evolution of PHS1, we first searched the presence of the tubulin kinase-like sequences. Kinase domains with high sequence similarity to the tubulin kinase domain were not found in the genomes of red algae. However, tubulin kinase domains were identified in many (but not all) genomes of green algae (Table 1). Every algae protein possessing a putative tubulin kinase had a Mitogen-activated Protein Kinase (MPK) phosphatase-like domain in its C-terminus. Therefore, these proteins share the overall domain architecture of PHS1.

**Table 1:**
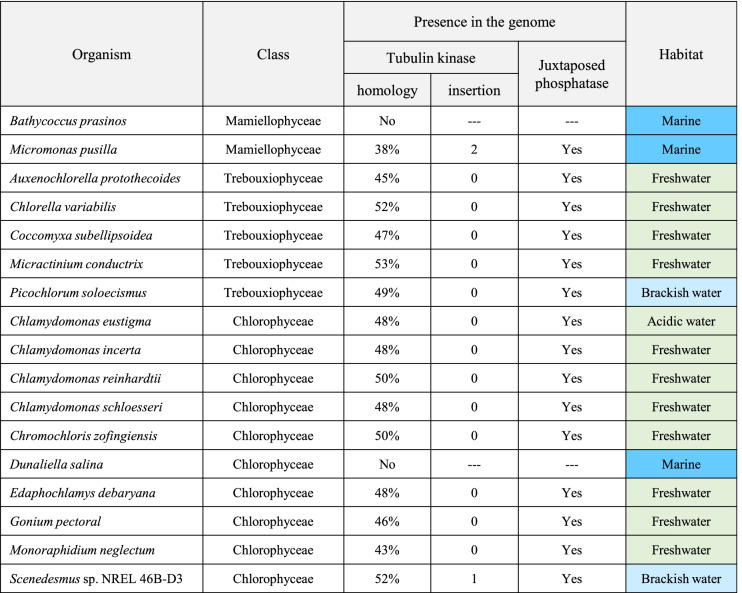
PHS1 homologues in the Chlorophyta green algae

In Chlorophyta, *PHS1* genes are present in nine Chlorophyceae species (*Chlamydomonas reinhardtii*, *Chlamydomonas eustigma*, *Chlamydomonas incerta*, *Chlamydomonas schloesseri*, *Chromochloris zofingiensis*, *Edaphochlamys debaryana,* *Gonium pectoral*, *Monoraphidium neglectum*, and *Scenedesmus* sp. NREL 46B-D3), five Trebouxiophyceae species (*Picochlorum soloecismus*, *Auxenochlorella protothecoides*, *Chlorella variabilis*, *Coccomyxa subellipsoidea*, and *Micractinium conductrix*), and one Maiellophyceae species (*Micromonas pusilla*). These green algae live in freshwater except for *P. soloecismus* (brackish water), *Scenedesmus* sp. (brackish water), and *M. pusilla* (marine). The tubulin kinase domains in the predicted PHS1 proteins in the *S.* sp. and *M. pusilla* contain insertions (105 amino acid residues in *Scenedesmus* sp., and 17 and 41 amino acid residues in *M. pusilla*) that are not found in other algal PHS1 sequences (Fig. S6). It is noteworthy that two marine green algae, *Bathycoccus prasinos* (Maiellophyceae) and *Dunaliella salina* (Chlorophyceae), do not possess any *PHS1*-like genes in their genomes. Possible correlation between the presence of PHS1 and the non-saline habitat is discussed below.

## Discussion

### *PHS1* genes in the plant lineage

A characteristic feature of the PHS1 structure is the presence of both a unique tubulin kinase domain and a MPK phosphatase-like domain in a single protein sequence. The tubulin kinase domain has a high sequence homology to the atypical kinase domain of the actin-fragmin kinases (which phosphorylate actin) found in slime molds, ciliates, and non-animal holozoans [17–19]. In PHS1, the tubulin kinase domain is situated between the N-terminal kinase interaction motif and the C-terminal phosphatase catalytic domain of a MPK phosphatase-like phosphatase. Since MPK phosphatases are ancient and are present all the eukaryotic organisms [20], insertion of the atypical kinase domain into a MPK phosphatase of an ancient early plants, probably by the horizontal gene transfer from an ancient lower single-celled animal, likely represents a hallmark of the evolution of the plant-specific PHS1 tubulin kinase.

All the sequenced genomes of Rhodophyta (red algae) lack *PHS1*, whereas many (but not all) green algae (the Chlorophyta division) and all the subsequently evolved plants possess genes encoding PHS1 homologues, indicating that *PHS1* evolved in Chlorophyta. The Chlorophyta green algae live in diverse environments, including marine, brackish water, and freshwater. Notably, all the freshwater green algae for which genomes are sequenced (including the Chlorophyceae class) possess apparently intact *PHS1* genes, while the two marine green algae, *Bathycoccus prasinos* (the Mamiellophyceae class) and *Dunaliella salina* (the Chlorophyceae class), do not show evidence of *PHS1* in their genomes. Since PHS1 is activated under the moderate salinity (such as 10% of sea water), marine habitat with constantly high salinity and high osmotic pressure may be detrimental to organisms possessing PHS1. We speculate that *PHS1* genes may have been lost in the marine-living organisms. A possible exception is the marine green algae *Micromonas pusilla* (the Mamiellophyceae class), in which a full length PHS1 is apparently encoded in its genome. We noted that two insertions disrupt the *Micromonas* kinase domain, raising a possibility that the tubulin kinase activity is affected by these insertions. A long insertion likewise disrupt the kinase domain of brackish water-living *Scenedesmus* sp. Tubulin phosphorylation activities of recombinant PHS1 kinase domains from *Micromonas* and the green algae of the brackish water habitat (*P. soloecismus* and *Scenedesmus* sp.) need to be examined to show whether these green algae PHS1 proteins possess functional tubulin kinase activities or not. As more genome sequences of green algae become available, possible association of PHS1 with freshwater habit will be more adequately addressed.

### Activation of PHS1 tubulin kinase

Thr349 of α-tubulin is specifically phosphorylated by *Arabidopsis* PHS1 *in planta* [3]. Phosphoproteomics in *Arabidopsis* seedlings revealed that this PHS1-target site is not or barely phosphorylated under unstressed conditions, but becomes increasingly phosphorylated after seedlings are dehydrated for up to 90 min [21] or are treated 5 min by 0.3 M mannitol or 0.15 M NaCl [22]. Phosphorylation of rice α-tubulins also becomes apparent after 5 min of 0.4 M NaCl or 0.8 M sorbitol treatment of rice seedling roots [23]. Immunohistochemical analysis of *Arabidopsis* leaf epidermal cells showed that interphase cortical microtubules become more disassembled after 10 min of 0.8 M sorbitol treatment, concomitant with increase of tubulin phosphorylation [3]. These results demonstrate that PHS1-mediated tubulin phosphorylation in angiosperms starts ca. 5 min after hyperosmotic stress and results in destabilization of interphase microtubule arrays.

In *Chlamydomonas*, we showed that PHS1 phosphorylates Thr349 of α-tubulin 10 min after application of > 0.2 M (i.e., > 200 mosM) sorbitol in both G1 phase and mitotic cells, and causes fragmentation of cytoplasmic microtubules after 10 min. Since the cytosolic osmolarity of *Chlamydomonas* cells is approximately 170 mosM [24], this response is caused by mild hyperosmotic stress. Although mitotic microtubule arrays were not examined in our study, due to technical difficulties to precisely define dense mitotic microtubule structures, mitotic arrays are likely destabilized by rapid tubulin phosphorylation. It appears that activation of PHS1 tubulin kinase activity occurs both in G1 phase and mitotic cells immediately upon hyperosmotic stress.

We showed that stress-induced tubulin phosphorylation is transient, even when osmotic stress persists; in the continuous presence of > 0.2 M sorbitol, phosphorylated *Chlamydomonas* tubulins are mostly de-phosphorylated after 1 h in G1 phase cells and after 3 h in mitotic cells. We do not know what causes the observed differences in the steady-state levels of phosphorylated tubulins under continuous stress during the cell cycle. De-activation (or de-sensitization) of the PHS1 tubulin kinase, as commonly observed for stress-activated MPKs [25], increased activity of putative tubulin de-phosphatases during continuous stress, or both, may be responsible for this phenomena.

Previous studies showed that Thr349 of α-tubulin is phosphorylated by stresses caused either by salt (NaCl) or osmotic agents (mannitol or sorbitol) [3, 22, 23]. Since salt stress to plants involves both ionic imbalance and hyperosmotic effects [26], we compared the effects of NaCl and sorbitol at the equivalent morality, and found that tubulin phosphorylation is mainly triggered by hyperosmotic stress, whereas NaCl inhibits cell growth strongly by ionic imbalance. Sensing mechanisms of salinity and osmotic stress are distinct in plants, but the molecular identities of responsible sensors and receptors are still largely elusive [27].

### Hyperosmotic stress responses in *Chlamydomonas*

Responses of *Chlamydomonas* cells to salinity stress (a combination of hyperosmotic and ionic stresses) have been studied (reviewed in [28]), and include alterations in morphology [29], photobiology [30], transcriptomes [31], proteomes [32], and plus-end dynamics of cytoplasmic microtubules [33]. Relevant studies on hyperosmotic stress are very limited; to our knowledge, only one study reports metabolomics changes of *Chlamydomonas* cells to hyperosmotic stress after 5 h [34].

We found that hyperosmotic stress causes PHS1-mediated transient tubulin phosphorylation and microtubule destabilization, as well as inhibition of daughter cell release after cell division. Newly divided daughter cells in *Chlamydomonas* and related green algae are encapsulated in the mucilage rich in acidic polysaccharides [35] and hydroxyproline-containing glycoproteins [36]. Various environmental stresses [37], including salinity (e.g., [38]), inhibit hatching, resulting in formation of daughter cell aggregates, called “palmelloids”. Although physiological roles of palmelloid formation are not well known, it has been proposed that formation of large cell aggregates enables the algal cells to better escape herbivory from grazing zooplanktons [39].

### Possible roles of PHS1-mediated microtubule destabilization

The spindle assembly checkpoint (SAC) functions as a surveillance mechanisms to ensure equal segregation of chromosomes during cell division, by arresting metaphase until all chromosomes are correctly attached to spindle microtubules [40]. Land plants and green algae possess both shared and unique cell cycle control components, compared with yeast and animals [41], and display compromised SAC responses. When spindle microtubules were not formed by treating *Arabidopsis* roots with a high dosage (1 μM) of oryzalin, a microtubule depolymerizing drug, metaphase cells were transiently arrested by SAC but re-initiated the cell cycle after ~ 2 h without subsequent cell division to attain polyploidy [42]. Such short-lived SAC functions during severe stress may have promoted frequent genome duplication during plant genome evolution [42]. DNA re-replication was also observed in *Chlamydomonas* cells when treated with a high dosage of the microtubule-depolymerizing drug amiprophos-methyl for a prolonged duration or disrupted in genes involved in tubulin folding chaperones or a microtubule nucleation [43]. Thus, severe disruption of mitotic microtubules in land plants and green algae does not abolish progression of the cell cycle due to transient SAC functions.

Our studies reveal that PHS1 might play a role in delaying cell cycle progression in *Chlamydomonas* under mild osmotic stress conditions. Moderate destabilization of mitotic microtubules by lower dosages of oryzalin (< 0.2 μM) delayed the progression of mitosis in *Arabidopsis* roots in a SAC-dependent manner [42]. Hyperosmotic stress by 0.3 M sorbitol might cause sufficient microtubule destabilization that would result in the cell cycle delay in *Chlamydomonas* cells. It will be interesting to study whether PHS1-mediated microtubule destabilization directly or indirectly causes the cell cycle delay, and whether the stress-responsive cell cycle regulation requires canonical SAC functions. Also, long-term effects of moderate hyperosmotic stress on chromosome segregation errors and genome instability worth investigation.

During G1 phase, hyperosmotic stress-triggered transient microtubule destabilization does not affect cell growth (the rate of cell volume increase). What roles might the PHS1-mediated microtubule destabilization have for cellular responses to hyperosmotic stress in the G1 phase algal cells? Future studies need to address whether cell behavior and motility, such as chemotaxis and phototaxis, are affected by the stress.

## Conclusion

In freshwater green algae *Chlamydomonas reinhardtii*, PHS1 tubulin kinase was activated by mild hyperosmotic stress and rapidly and transiently phosphorylated α-tubulin at a critical Thr residue required for efficient polymerization. Tubulin phosphorylation resulted in transient disassembly of interphase microtubules. Hyperosmotic stress-induced transient tubulin phosphorylation delayed progression of mitosis, and is thought to be partly responsible for stress-responsive cell cycle delay. Presence of *PHS1* homologues in many freshwater living green algae indicate their possible roles in salinity response.

## Methods

### Strains, media and culturing conditions

*C. reinhardtii* wild-type strain CC-4533 and *PHS1* insertion mutant LMJ.RY0402.181368 (*phs1–1*) were obtained from Chlamydomonas Library Project (https://www.chlamylibrary.org/ [44];). *Chlamydomonas* cells were maintained and cultured in the Tris-acetate-phosphate (TAP) medium (0.4 g L^− 1^ NH_4_Cl, 0.1 g/L MgSO_4_·7H_2_O, 0.05 g L^− 1^ CaCl_2_·2H_2_O, 0.108 g L^− 1^ K_2_HPO_4_. 0.056 g L^− 1^ KH_2_PO_4_, 2.42 g L^− 1^ Tris, 1 mL L^− 1^ glacial acetic acid, 5 mg L^− 1^ Na_2_·EDTA, 2.2 mg L^− 1^ ZnSO_4_·7H_2_O. 1.14 mg L^− 1^ H_3_BO_3_, 5.1 mg L^− 1^ MnCl_2_·4H_2_O, 0.5 mg L^− 1^ FeSO_4_·7H_2_O, 0.16 mg L^− 1^ CoCl_2_·6H_2_O, 0.16 mg L^− 1^ CuSO_4_·5H_2_O, 0.11 mg L^− 1^ (NH_4_)_6_Mo_7_O_24_·4H_2_O and 1.6 mg L^− 1^ KOH). Stock solutions of 3 M NaCl and 3 M sorbitol were diluted prior to each experiment to give indicated final concentrations. A commercial seawater mixture (GEX Co., Osaka, Japan) was dissolved in sterilized water to prepare artificial seawater.

To synchronize cell growth, cells were grown in 25 mL of the TAP liquid medium with horizontal shaking at 100 rpm in an incubator shaker under white light (224 μmol photons m^− 2^ s^− 1^) with a light:dark (12 h:12 h) photoperiod. Prior to the start of the synchronized culture, cells were diluted to OD_750_ = 0.05 at the beginning of the light regime for several days. Approximately 2 × 10^5^ cells were inoculated into 25 mL medium. The temperature in the incubator shaker was set at 26 °C during illumination and at 22 °C during the dark regime.

### Search for *PHS1* homologues

The *Arabidopsis thaliana* PHS1 protein sequence (NP_851066.2) was used as a query against genome sequences deposited in Phytozome, Phycocosm, ConGenIE and GenBank at National Center for Biotechnology Information (NCBI). *PHS1* homologues were identified with two criteria: (a) a tubulin kinase domain (which contains three catalytically critical amino acid residues, corresponding to K187, D309, and N324 in *A. thaliana* PHS1) juxtaposed with a MAP kinase phosphatase domain in a single ORF, and (b) confidence value/Expect (E)-value less than e^− 10^ of the kinase domain.

### Cell number and cell size analysis

The Z2 Cell and Particle Counter (Beckman Coulter) was used to measure the number of cells and the cell volume. Cells in the sampled 100 μL culture were first fixed with 1% glutaraldehyde and then immediately mixed with 10 mL of Isoton II diluent (Beckman Coulter). The cell size range was set between 60 to 3400 μm^3^. Data was exported using Coulter Z2 AccuComp. (Beckman Coulter).

Statistical significance in growth rate was calculated by one-way ANOVA (Analysis of Variance) with Tukey HSD (Tukey Honest Significant Differences) post-hoc test, using R statistical software ver3.6.3.

### Plasmid construction and transformation

A hygromycin selectable marker [45] was cloned between the *EcoR*V and *Kpn*I sites of *pBluescript II KS* (+) plasmid. A genomic fragment of *PHS1* (11,063 bp) was released from the fosmid clone (GCRFno27_g18 from the *C. reinhardtii* C9 strain) by digestion with *Hind*III and cloned into the *Hind*III site of the above *pBluescript II* plasmid to generate pgCrPHS1.

Transformation was performed according to [46] with some modifications. Fresh overnight culture of *Chlamydomonas* cells were centrifuged at 600 x *g* for 5 min and washed twice with 10 mL of MAX Efficiency Transformation Reagent for Algae (Invitrogen). The 1 × 10^8^ cells and approximately 1 μg of the *XbaI*-restricted pgCrPHS1 plasmid were mixed and then transferred to an electroporation cuvette. After incubation for 5 min at 4 °C, a single electric pulse with voltage of 0.4 kV for about 4 s was applied using MicroPulser™ (Bio-Rad). Electroporated cells were transferred to 10 mL of TAP medium containing 40 mM sucrose and were grown under dim light overnight. The cells were then spread onto TAP agar plate containing 30 μg mL^− 1^ hygromycin, and grown for 5 days. Putative transformants were picked and checked by genomic PCR analysis.

### DNA extraction and polymerase chain reaction (PCR)

*Chlamydomonas* cells grown on a TAP agar plate were mixed well in 50 μL of 5% Chelex (Invitrogen) and were vortexed for 15 s before subjected to 100 °C for 10 min. The tube was then placed on ice for 1 min and centrifuged at 12,000 rpm for 2 min. The supernatant was transferred to a new tube and was used for PCR analysis. Each sample contained a final concentration of 1 x PCR buffer for KOD FX Neo, 0.4 mM dNTP, 0.2 μM PCR forward and reverse primers, 0.5 μL KOD FX Neo, 0.5 μL of DNA template, and sterile water to fill up to 25 μL. PCR conditions: 95 °C for 5 min; followed by 30 cycles of 95 °C for 5 s, 58 °C for 45 s and 72 °C for 1 min, and finally 72 °C for 2 min. The primers sequences: PHS1_P1, 5′-CACACATAGGTGACAATGGGACAC-3′, PHS1_P2, 5′-GCAGCAGTAGCAACATAAGCAGTA-3′ and LB, 5′-TGGGCGCCGTAGTTAAGACAAA-3′.

### Quantitative real-time PCR

*Chlamydomonas* cells were harvested by centrifugation at 600 x *g* for 5 min and disrupted by mortar and pestle with liquid nitrogen. Total RNA was extracted by using RNeasy Mini kit (Invitrogen) according to the manufacturer’s instructions. Cell homogenates were mixed with 450 μL of the RLT buffer and transferred to a QIA shredder spin column. After centrifugation, the flow-through fraction was collected and 225 μL of 100% ethanol was added. The mixture was transferred to an RNeasy spin column. After centrifugation at 8000 x *g* for 15 s, 700 μL of RW1 buffer was added to the column and subjected to centrifugation at 8000 x *g* for 15 s. The flow-through fraction was discarded. A total of 500 μL RPE buffer was added to the column and again centrifuged at 8000 x *g* for 15 s. This step was repeated using 500 μL RPE buffer but with centrifugation for 2 min. The column was carefully transfer to a new 1.5-mL centrifuge tube. Finally, 30 μL of RNase free water was added into the column and centrifuged at 10,000 x *g* for 1 min and kept at − 80 °C until use.

ReverTraAce®qPCR MasterMix with gDNA remover (Toyobo) was used to reverse transcript RNA. A total of 0.5 μg of RNA was used per reaction. All samples were heated to 65 °C for 5 min and transferred to ice immediately. Then, 4 x DN master mix was added. After the samples were incubated for 37 °C for 5 min, 5 x RT master mix was added into the reaction mixture and then subjected for incubation: 37 °C for 15 min, 50 °C 5 min, 98 °C for 5 min. The cDNA preparations were kept at − 20 °C until use.

RT-qPCR was performed using Light Cycler 96 (Roche) with TB Green™ *Premix Ex Taq*™ II (Takara). Each sample consists of a final concentration of 1 x TB Green Premix Ex Taq II, 0.4 μM of PCR forward and reverse primers, and 1 μL of cDNA template top up to 10 μL. The thermal program was 95 °C for 30 s, followed by 40 cycles of 95 °C for 5 s and 60 °C for 30 s, 1 cycle of 95 °C for 5 s, 60 °C for 1 min, and finally 50 °C for 30 s. A sodium/phosphate symporter gene (*PTB-1)* was used as a reference gene. Primers sequences for qPCR: PTB1-F, 5′-GCCTACTCGCCCAGCATC-3′, and PTB1-R, 5′-TGTTGGTGCGGTTGAGCA-3′; CDKB-F, 5′-ACCTGCACCGCATCTTCC-3′, and CDKB-R 5′-GGGTGGTTGATCGCCTCC-3′; CYCB-F, 5′-TGCCCAGCGACTACATGAC-3′, and CYCB-R, 5′-CGTCTCGGGCATCAGCTT-3′.

### Protein extraction

*Chlamydomonas* cells were harvested by centrifugation at 600 x *g* for 5 min*,* quick frozen in liquid nitrogen, and crushed with mortar and pestle. Protein extraction buffer [20 mM sodium phosphate buffer (pH 7.4) containing 100 μM Na_3_VO_4_, 50 mM β-glycerophosphate, 0.5 mM phenylmethanesulfonylfluoride and one tablet of Complete Protease Inhibitor Cocktail (Roche)] was used to re-suspend the cells. After centrifugation at 15,000 x *g* for 20 min, the cell extracts were transferred to a new tube, added with 3 times volume of acetone, and incubated overnight at − 20 °C. The protein extract was centrifuged at 15,000 x *g* for 10 min and re-suspended in 1 x sample buffer (62.5 mM Tris-HCl, pH 6.8, 2.5% SDS, 0.002% bromophenol, 10% glycerol). The protein concentration was measured using RC DC Protein Assay (Bio-rad) by using bovine serum albumin as a control. The absorbance readings were taken using Ultrospec 3000 UV/Visible spectrophotometer (Pharmacia Biotech) at 750 nm.

### Immunoblotting

Proteins (20 μg) were resolved on 10% SDS-PAGE (Resolving gel: 375 mM Tris-HCl, 10% acrylamide, 0.1% SDS, 0.1% APS and 0.04% TEMED; stacking gel: 147 mM Tris-HCl, 5% acrylamide, 0.1% SDS, 0.1% APS and 0.1% TEMED) with a 1 x SDS running buffer (25 mM Tris, 192 mM glycine and 0.1% SDS). For phos-tag 10% SDS-PAGE, 50 mM MnCl and 25 μM Phos-tag (Wako) were added in addition of 10% SDS PAGE resolving gel mixture. The SDS PAGE electrophoresis were performed using voltage of 300 V, 40 mA for 100 min. After electrophoresis, the gel was washed in transfer buffer (25 mM Tris, 192 mM glycine and 10% methanol) for 5 min. Phos-tag gel was washed in the transfer buffer containing 10 mM EDTA. The proteins were then blotted onto a polyvinylidene fluoride (PVDF) membrane (Immobilon-P, Merck Milipore) and electrophoresis was performed using transfer buffer for 60 min at 100 V, 400 mA. After electrophoresis, the membrane was washed with TBS-T buffer (20 mM Tris, 150 mM NaCl and 0.1% Tween 20) before blocked with blocking buffer (5% skim milk in TBS-T) for 1 h. Then, the blotted membrane was incubated overnight with a primary antibody in the blocking buffer. Primary antibodies used in this study were anti-α-tubulin mouse antibody (B-5-1-2) (1:100,000 dilution) (Sigma) and anti-pT349 rabbit antibody (1:5000 dilution) [6]. After washing with TBS-T three times, the membrane was incubated with anti-mouse IgG, HRP-linked antibody (GE-Healthcare; 1:10,000 dilution) or anti-rabbit IgG, HRP-linked antibody (GE-Healthcare; 1:10,000 dilution) as secondary antibodies in the blocking buffer for 1 h. Immobilon western chemiluminescent HRP substrate (Milipore) was used for immunoblot detection. Chemiluminescence was detected using ImageQuant LAS4000 (GE Healthcare Life Sciences) and quantified by using Image J.

### Microscopy analyses

For immunofluorescence analysis, approximately 100 μL of *Chlamydomonas* cells were placed on a clean polylysine-coated microscopy slide, which had been prepared by treating the slide with 0.1 mg/mL of polylysine solution (Sigma-Aldrich) for 15 min. After immobilizing the cells on the polylysine slide for 5 min, 100 μL of PBS containing 4% (w/v) formaldehyde were applied to the cells for another 20 min. After washing with PBS three times, the slide was immersed in chilled methanol at − 20 °C for 20 min. The slide was washed with PBS three times again. Then, the slide was treated with 100 μL of blocking solution [5% (w/v) BSA in PBS-T buffer (PBS solution added with 0.1% of Tween 20)] for 1 h, and then with primary antibodies. For double-labeling immunofluorescence experiments, anti-α-tubulin rat antibody (YOL 1/34) (Milipore) and anti-acetylated α-tubulin mouse antibody (B-6-1-1)(Sigma) were used. All primary antibodies were applied at a dilution ratio of 1:2000. The slide was incubated at 4 °C overnight, washed with PBS-T for three times, and then incubated with goat anti-rat Alexa Fluor® 488 (Invitrogen) and goat anti-mouse Alexa Fluor® 568 (Invitrogen) with a dilution ratio of 1:200 for 1 h. After washing with PBS-T three times, the slide was treated with a mounting solution (100 mM Tris, 50% glycerol and 1 mg/mL para-phenylenediamine) and covered with 22 × 22 mm coverslip. Microscopy slides were examined with a Nikon Eclipse T*i*-U microscope equipped with a Yokogawa CSU-XI spinning disc confocal unit and Nikon 100x/1.30 oil immersion objective lens. Images were captured and recorded at 0.5 μm intervals at the wavelength of 488 nm and 568 nm by using Andor iXon3 DU897 EM-CCD camera. All images were documented by stacking using Z series. Image J was used to merge images of two chromophores, and the brightness was adjusted. Binary image of *Chlamydomonas* cells created by image J was used to evaluate the occupancy of microtubules in cell body over the cell size. Statistical significance between the means of group was calculated by one-way ANOVA with Tukey HSD post-hoc test, where *p*-value < 0.01 indicates significantly different.

For bright field microscopy, cells were treated with 1% glutaraldehyde and placed onto a microscope slide. Microscope slides were examined by using Nikon Eclipse E600 fluorescence microscope (Nikon) with Nikon 40x/0.75 objective lens. All mages were captured using Olympus DP70 camera and documented using DP controller software (Olympus).

## Supplementary Information

**Additional file 1: Figure S1.** Cell size distribution in a 12/12h of light/dark diurnal cycle. **Figure S2.** Growth (cell volume increase) of *Chlamydomonas* cells in the control medium, or in the culture medium containing 0.3 M sorbitol or 0.15 M NaCl. **Figure S3.** Immunoblots of tubulins in various *Chlamydomonas* strains. **Figure S4.** T-DNA insertion mutant of *PHS1*. **Figure S5.** Original fluorescence images and the corresponding binary images of microtubules generated by Image J. **Figure S6.** Amino acid sequence alignment of putative kinase domains from green algae PHS1 homologues.

**Additional file 2:.** Uncropped versions of immunoblots and an agarose gel.

## Data Availability

Materials generated in this work will be provided after reasonable requests to TH.

## Abbreviations

ABA: Abscisic acid
MPK: Mitogen-activated Protein Kinase
PCR: Polymerase chain reaction
PHS1: Propyzamide Hypersensitive 1
SAC: Spindle assembly checkpoint
SDS-PAGE: Sodium dodecyl sulfate-polyacrylamide gel electrophoresis
