# Supplementary material for: Hyperosmotic stress-induced microtubule disassembly in Chlamydomonas reinhardtii
Source: BMC Plant Biol. 2022 Jan 22;22:46. doi: 10.1186/s12870-022-03439-6 (PMC8783414; doi:10.1186/s12870-022-03439-6)
Supplement: Supplementary file 1 — Additional file 1: Figure S1. Cell size distribution in a 12/12h of light/dark diurnal cycle. Figure S2. Growth (cell volume increase) of Chlamydomonas cells in the control medium, or in the culture medium containing 0.3 M sorbitol or 0.15 M NaCl. Figure S3. Immunoblots of tubulins in various Chlamydomonas strains. Figure S4. T-DNA insertion mutant of PHS1. Figure S5. Original fluorescence images and the corresponding binary images of microtubules generated by Image J. Figure S6. Amino acid sequence alignment of putative kinase domains from green algae PHS1 homologues. [file 12870_2022_3439_MOESM1_ESM.pdf]

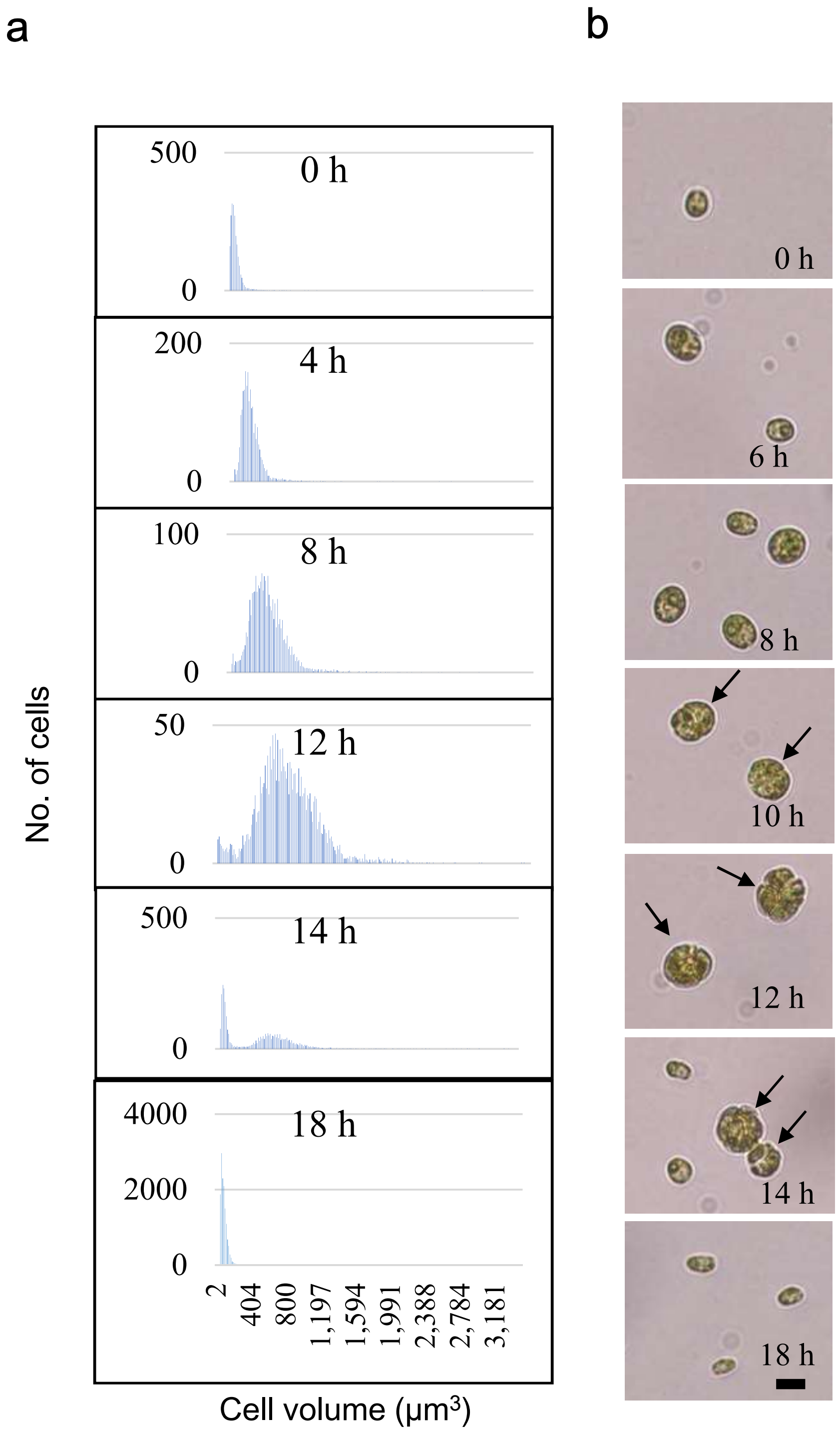

Figure S1: Cell size distribution in a 12h/12h of light/dark diurnal cycle. (**a**) Cell volume histograms at 0, 4, 8, 12, 14 and 18 h, (**b**) bright field microscopic images of *Chlamydomonas* cells at 0, 6, 8, 10, 12, 14 and 18 h. Arrows indicate clusters of newly divided daughter cells. Bar: 10 μm.

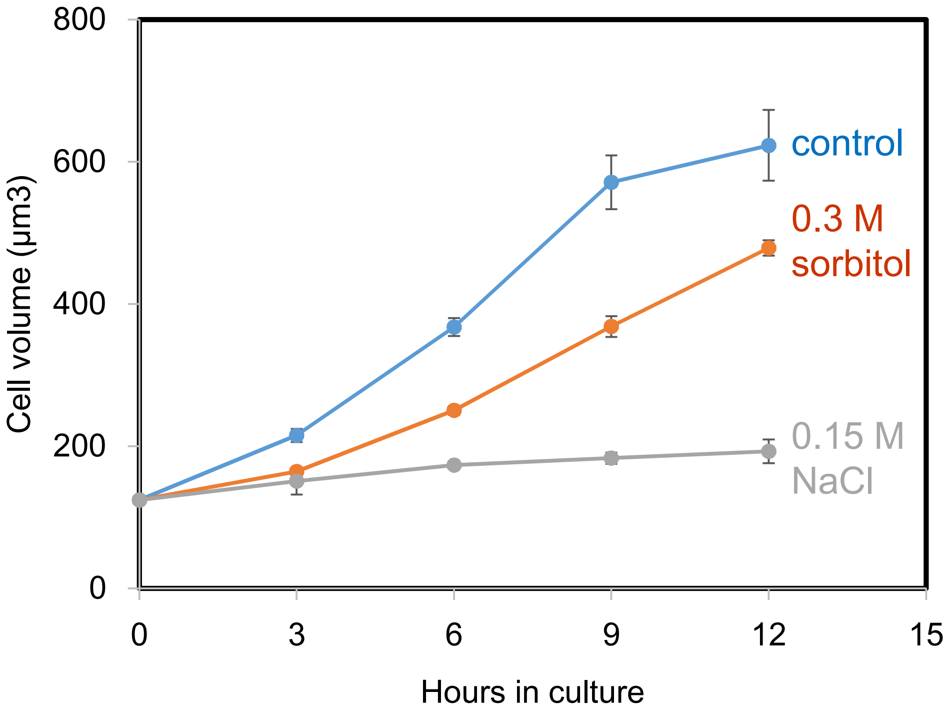

Figure S2. Growth (cell volume increase) of *Chlamydomonas* cells in the control medium, or in the culture medium containing 0.3 M sorbitol or 0.15 M NaCl. Error bars indicate standard deviations for three biological replicates.

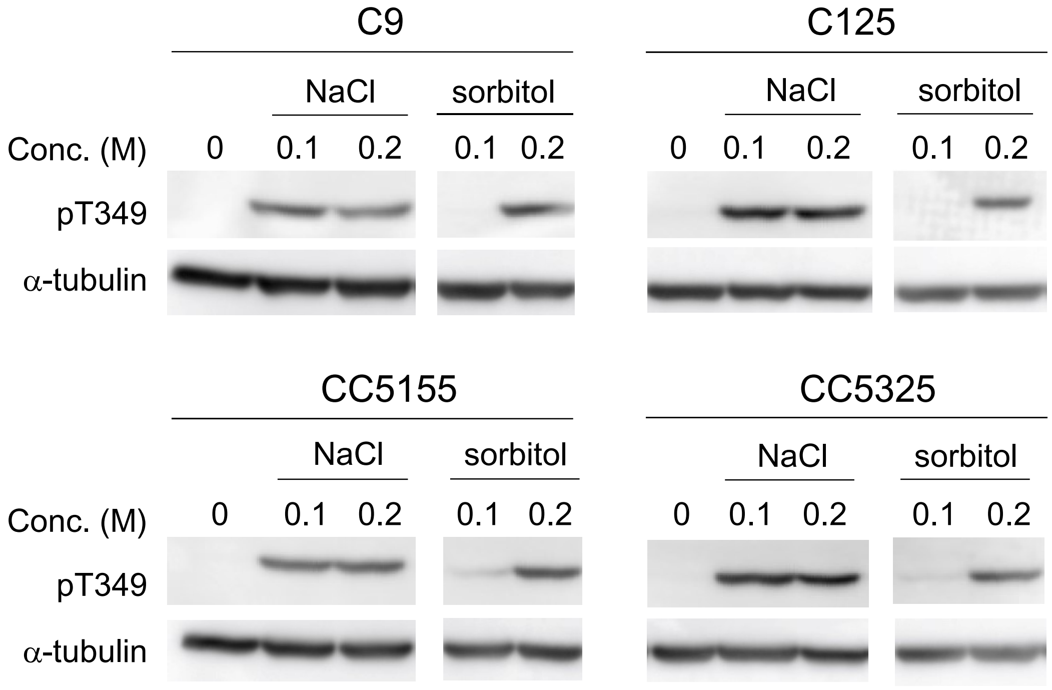

Figure S3. Immunoblots of tubulins in various *Chlamydomonas* strains. Cells were treated with NaCl or sorbitol at the indicated concentrations for 20 min. Proteins were separated by SDS-PAGE, blotted on membranes, and analyzed by anti-pT349 antibody or anti-α-tubulin antibody.

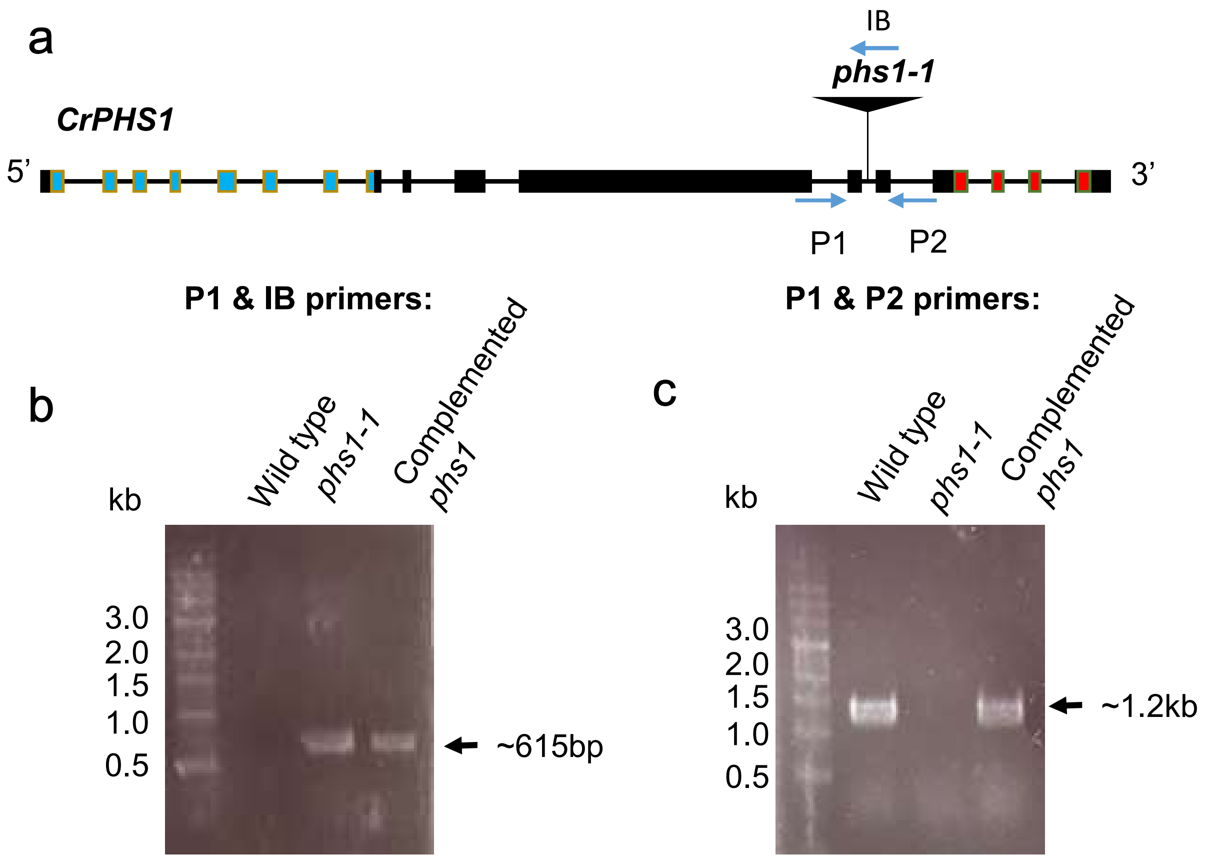

Figure S4. T-DNA insertion mutant of *PHS1*. (**a**) Gene structure of *PHS1*. The boxes and connecting lines indicate exons and introns, respectively. Tubulin kinase and phosphatase domains are respectively shown in blue and red boxes. Insertion site of the *aphVIII* gene in *phs1-1* is shown. Blue arrows indicate the primer locations for genomic PCR. (**b**, **c**) Agarose gel electrophoresis of *Chlamydomonas* genome fragments. Genomic DNAs from wild type, *phs1-1* and a complemented *phs1-1* line were amplified using the indicated primers targeting to probe the presence of the *aphVIII* gene (**a**) and intact *PHS1* gene (**b**).

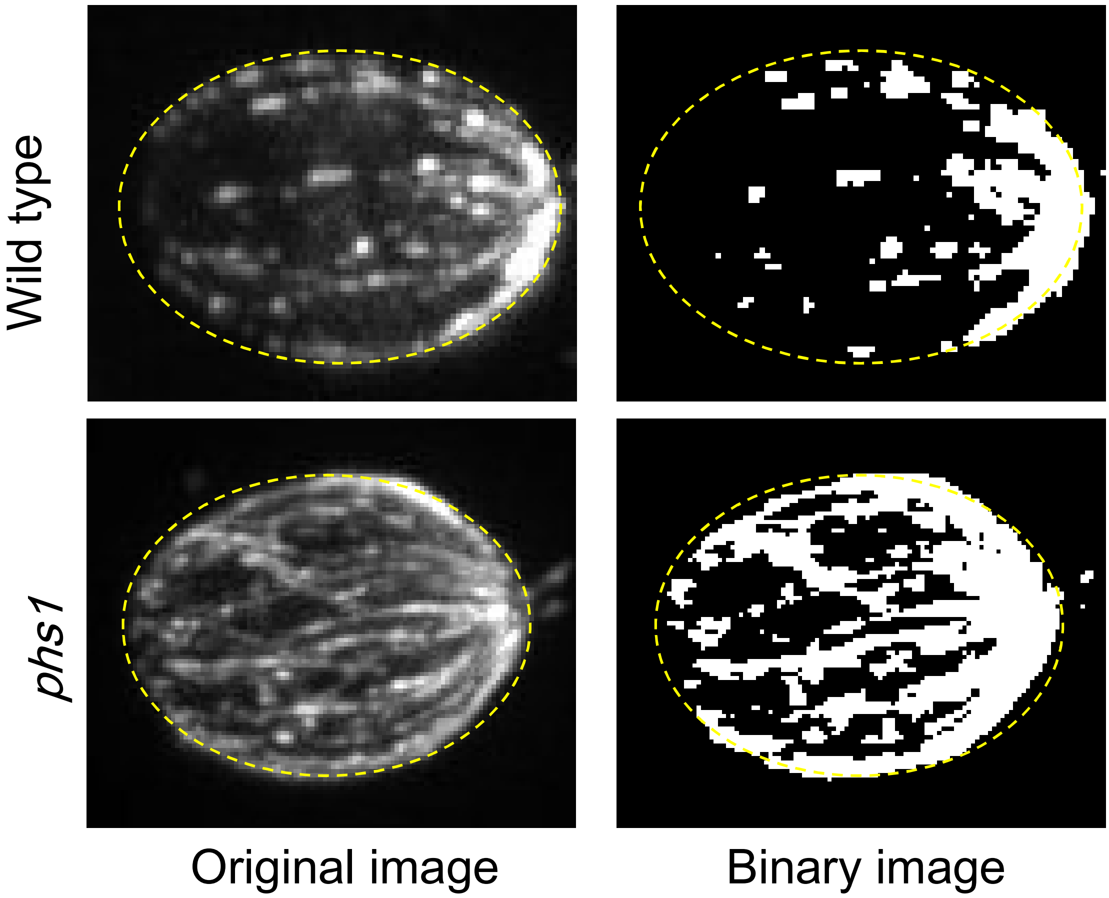

Figure S5. Original fluorescence images and the corresponding binary images of microtubules generated by Image J. *Chlamydomonas* cells were treated with 0.3 M sorbitol for 10 min, and microtubules were immunostained with anti-α-tubulin antibody. The cell contours are marked by yellow dashed lines. Microtubule occupancy was calculated by dividing the microtubule area by whole cell area.

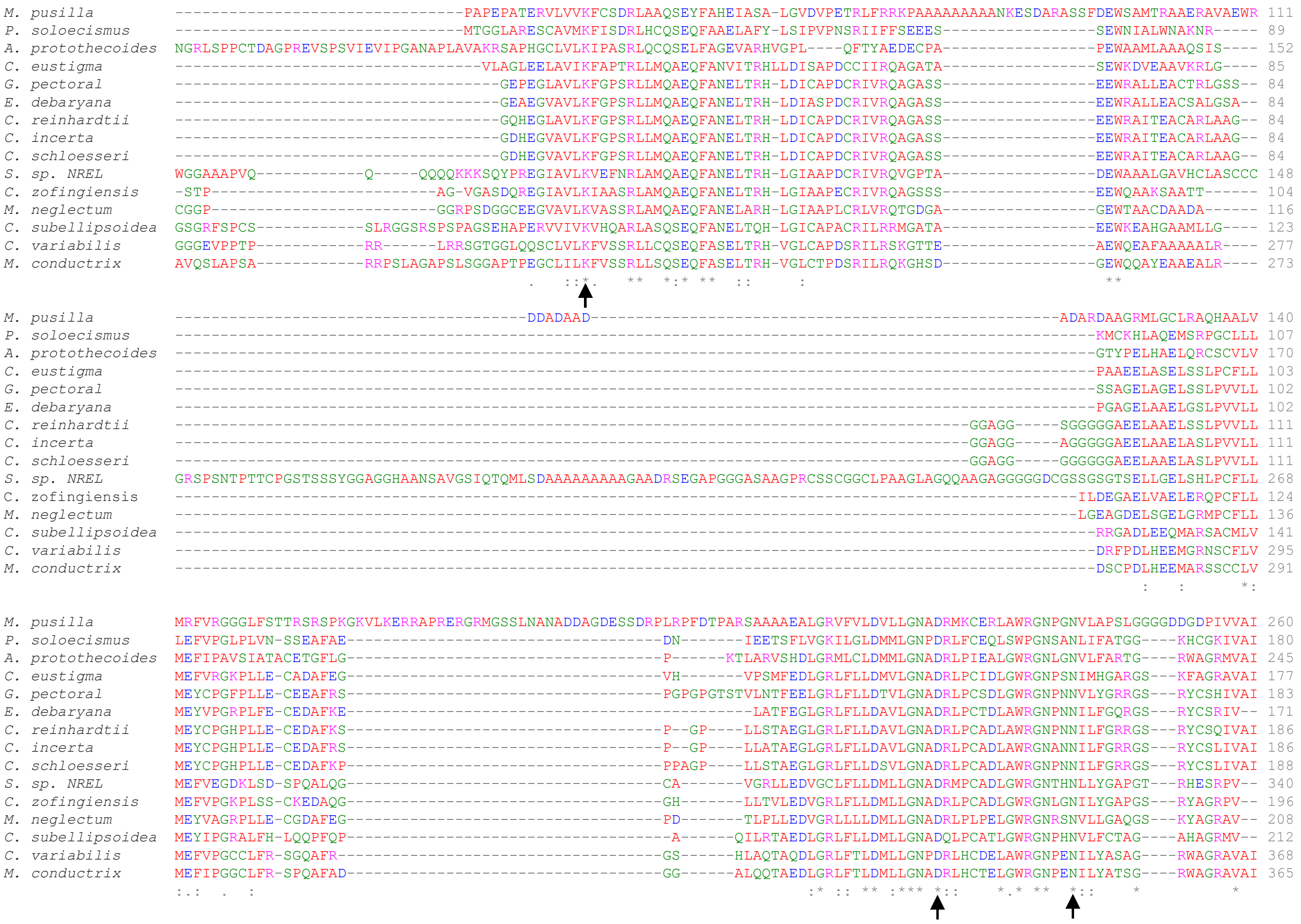

Figure S6. Amino acid sequence alignment of putative kinase domains from green algae PHS1 homologues. At the bottom of aligned sequences, invariant residues are indicated by asterisks, while conserved residues by dots. Three critical residues involved in ATP binding are indicated by arrows. Also, see Table 1 for full description of algal species.
