# Supplementary material for: Hyperosmotic stress-induced microtubule disassembly in Chlamydomonas reinhardtii
Source: BMC Plant Biol. 2022 Jan 22;22:46. doi: 10.1186/s12870-022-03439-6 (PMC8783414; doi:10.1186/s12870-022-03439-6)
Supplement: Supplementary file 2 — Additional file 2:. Uncropped versions of immunoblots and an agarose gel. [file 12870_2022_3439_MOESM2_ESM.pdf]

Figure 2A

pT349

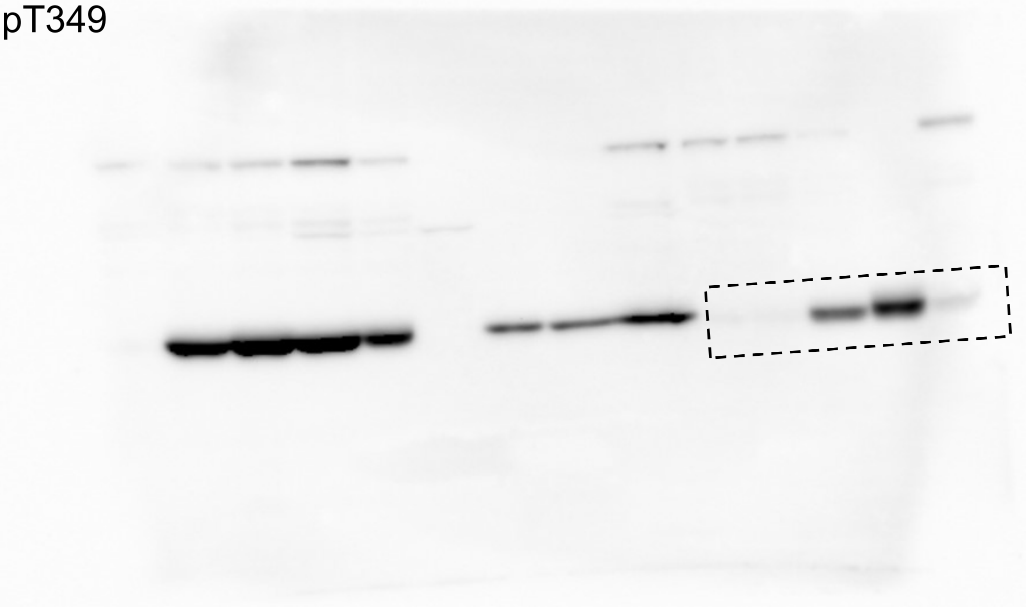

α-tubulin

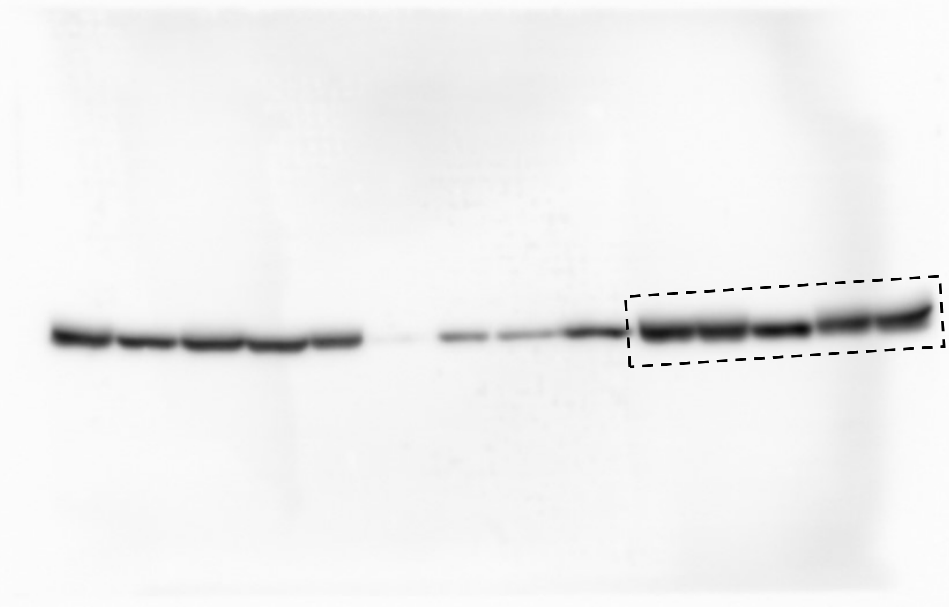

Figure 2B

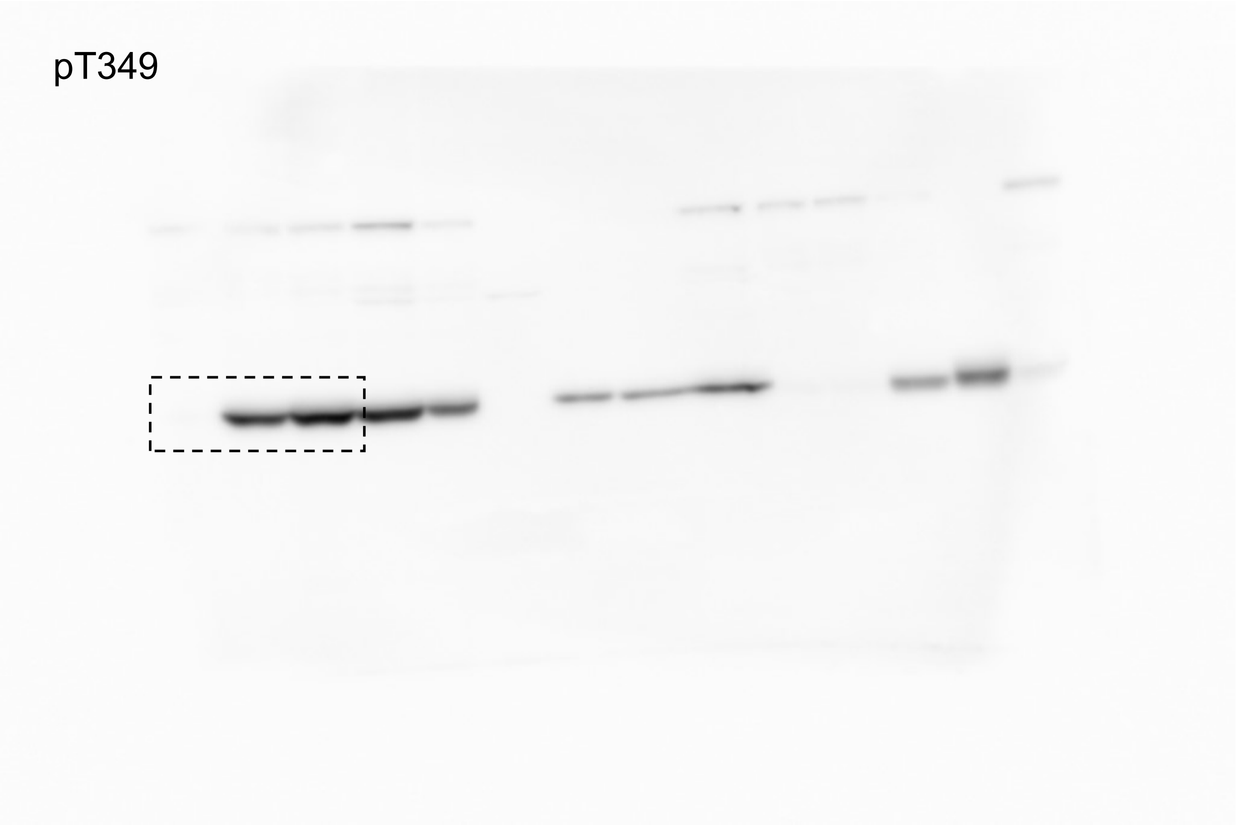

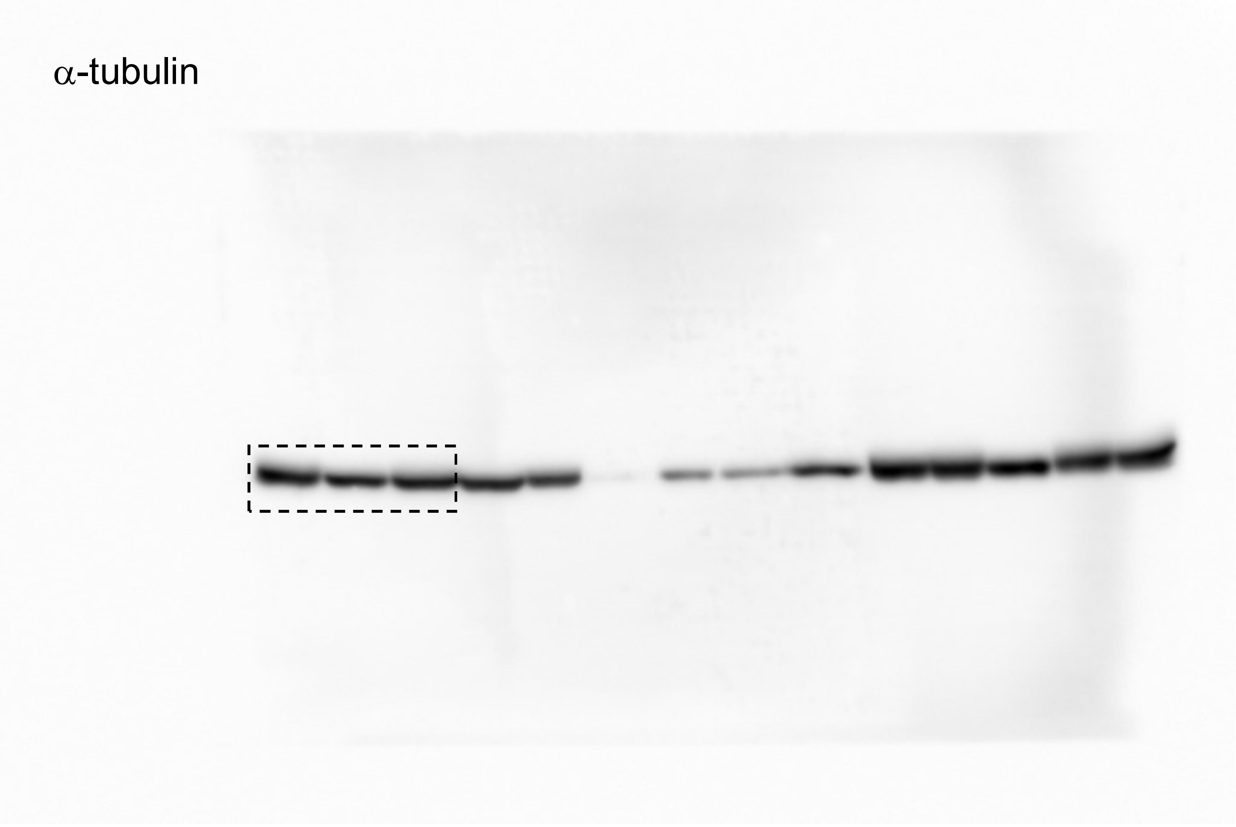

Figure 2D

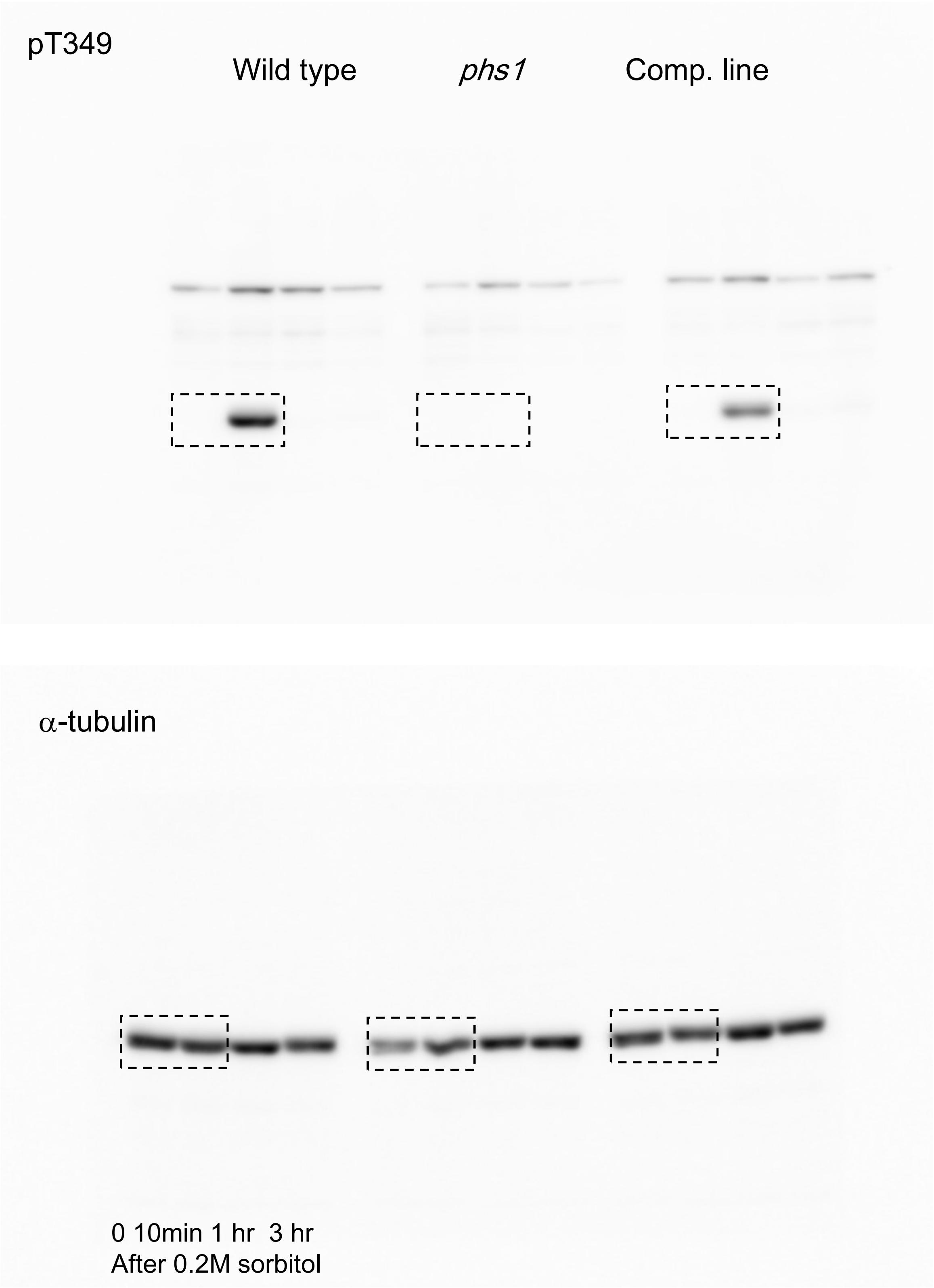
pT349
Wild type
phs1
Comp. line
α-tubulin
0 10min 1 hr 3 hr
After 0.2M sorbitol

Figure 3A

0.2 M sorbitol G1

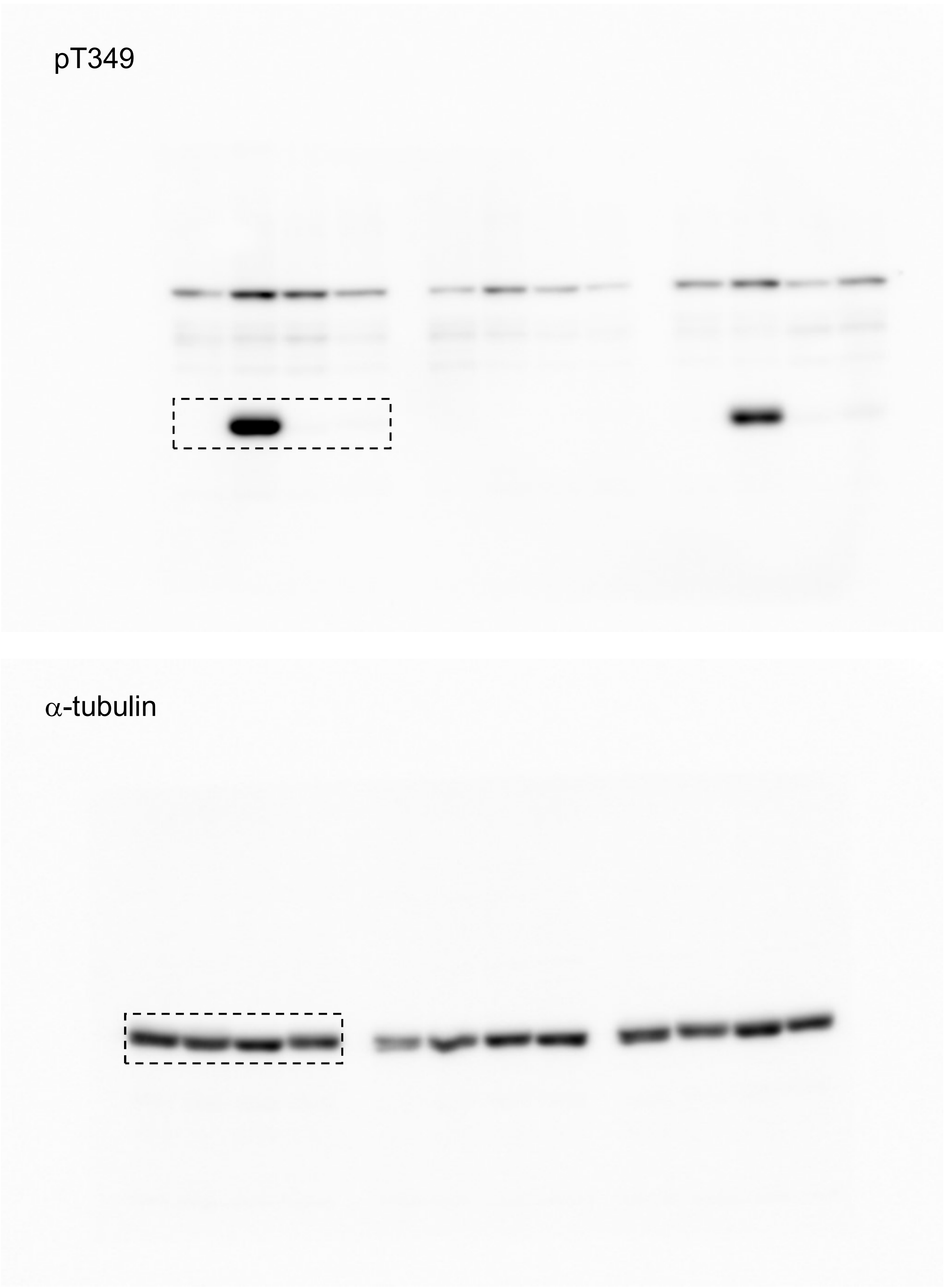

Figure 3A

0.3 M sorbitol G1

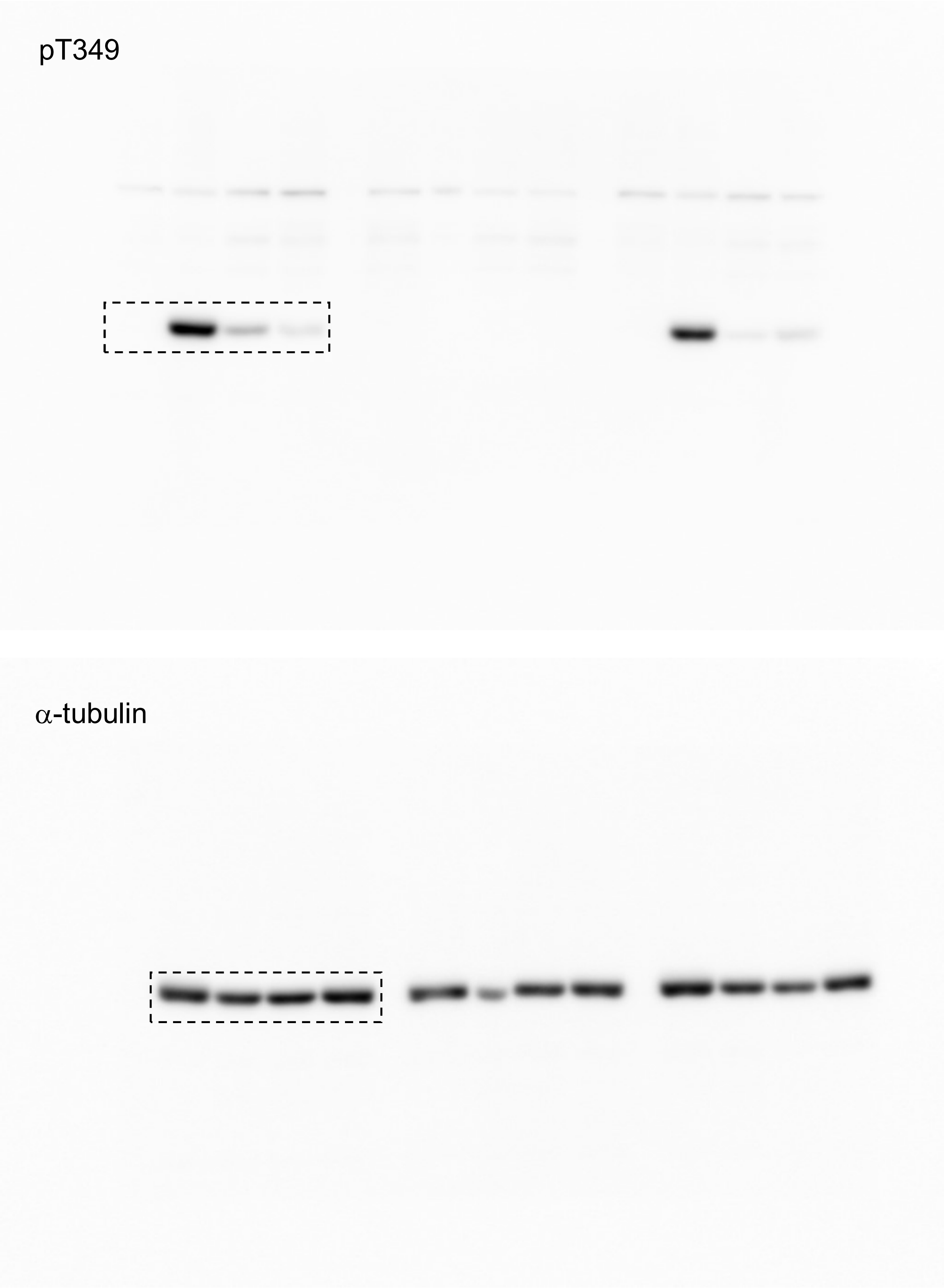

Figure 3A mitosis

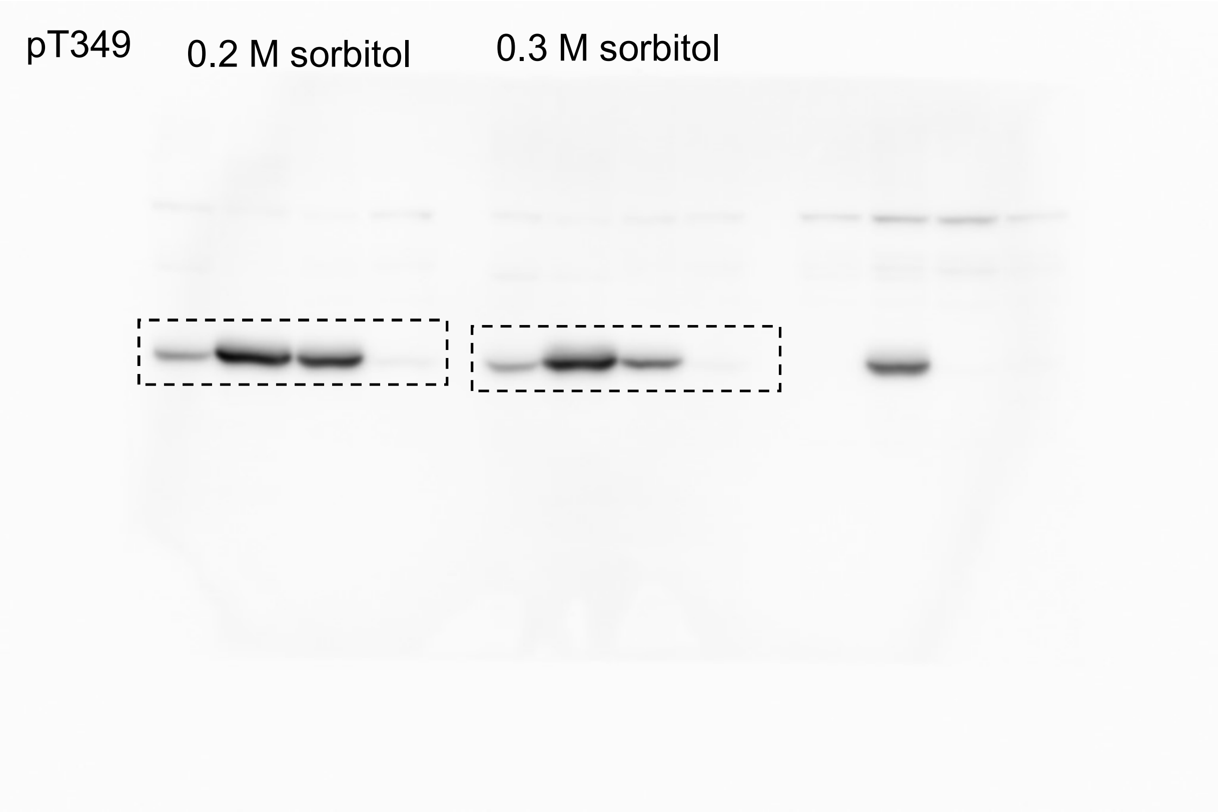

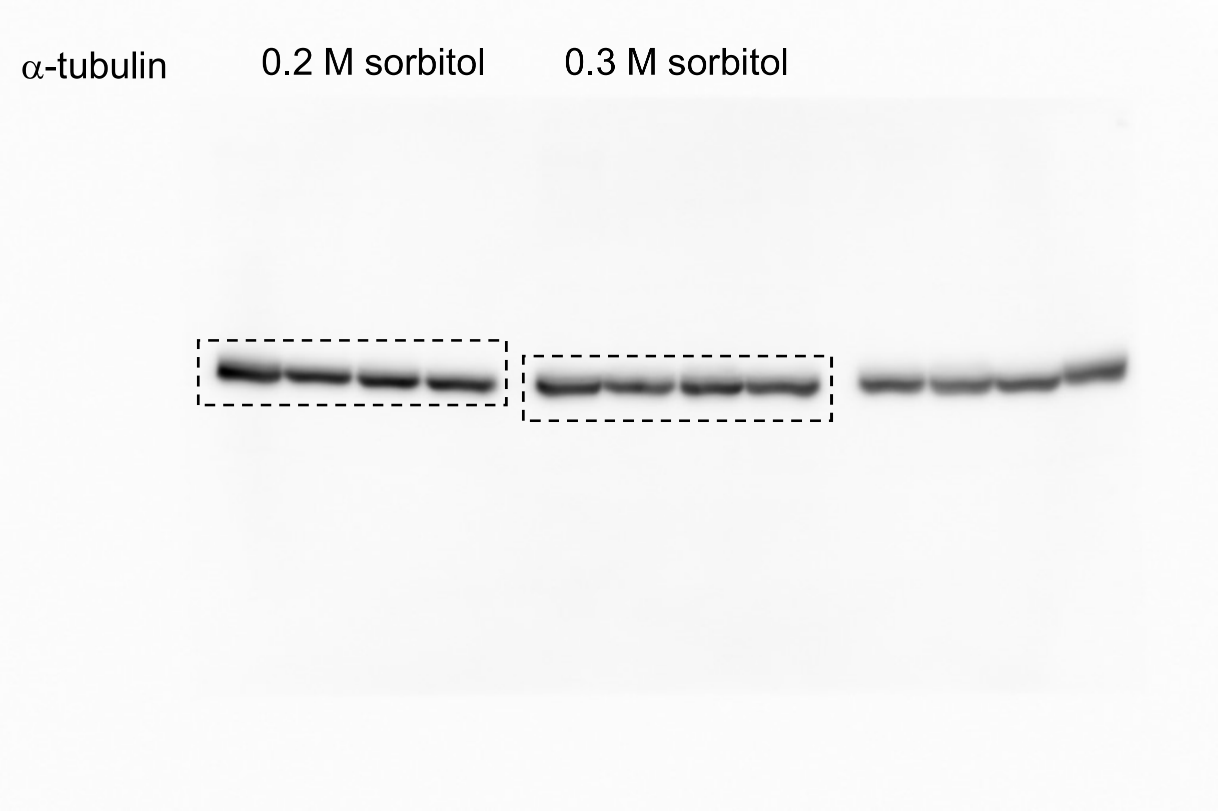

Figure 3B phostag

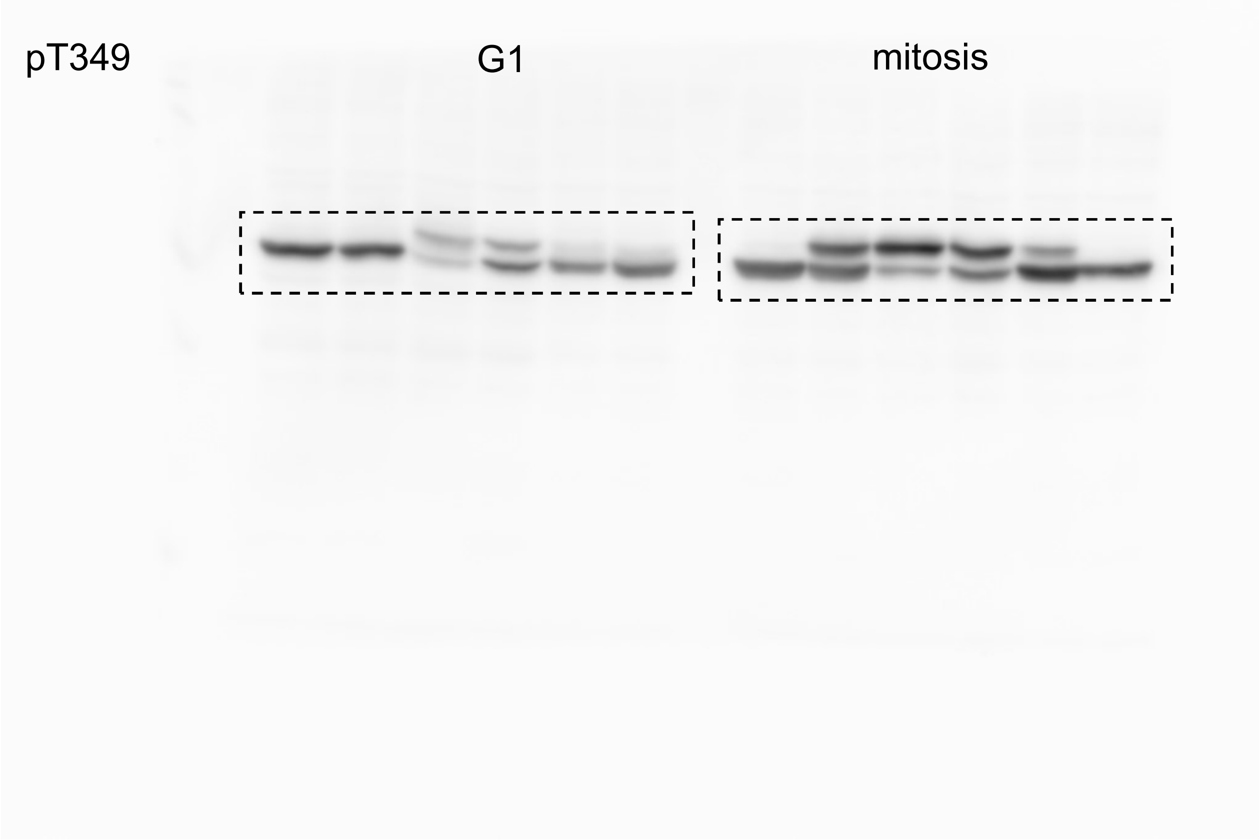

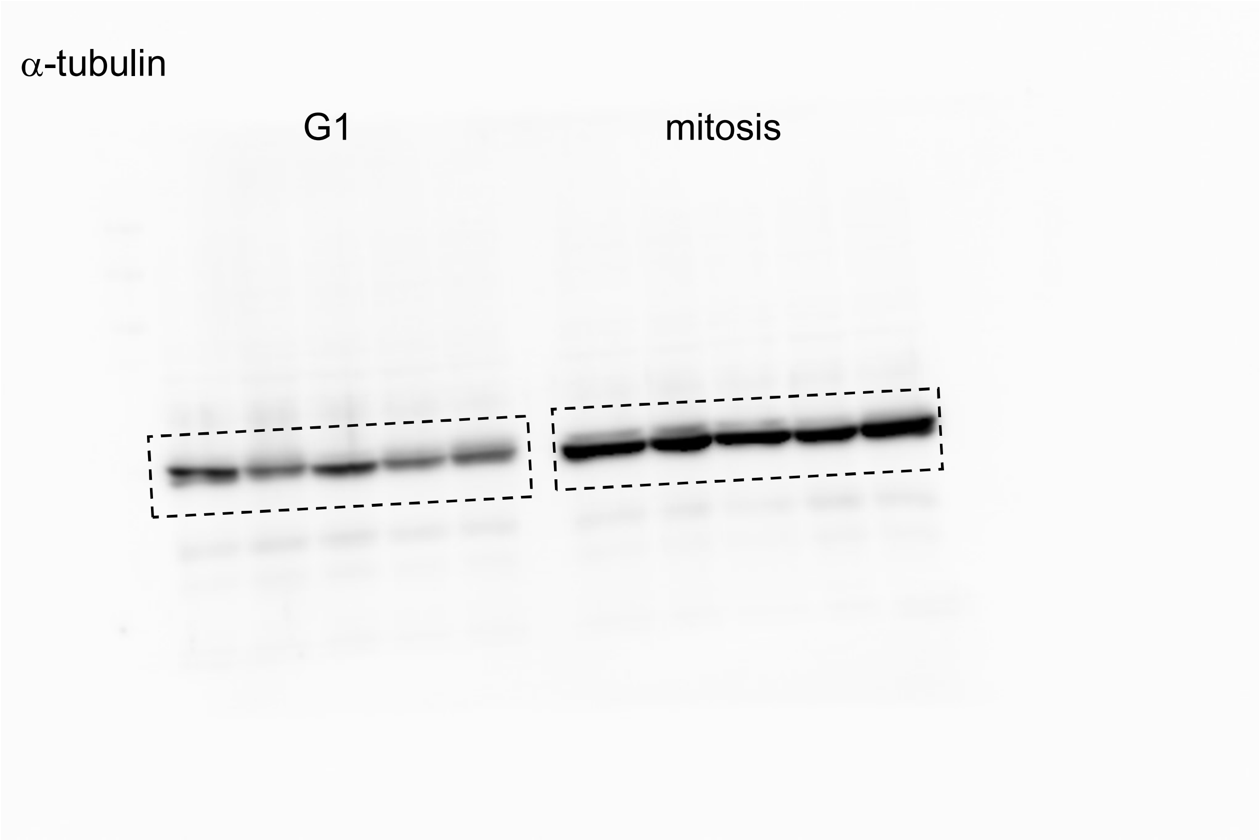

Figure S3

The appropriate portions of five SDS-PAGE gels were excised and blotted onto one membrane.

pT349

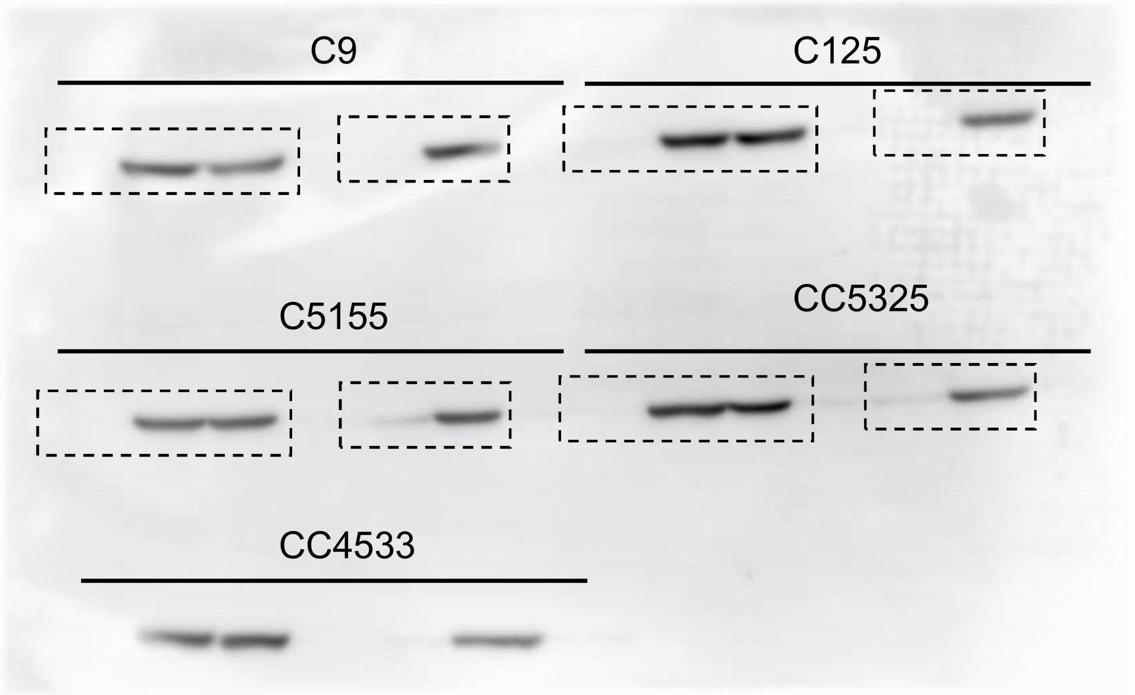

α-tubulin

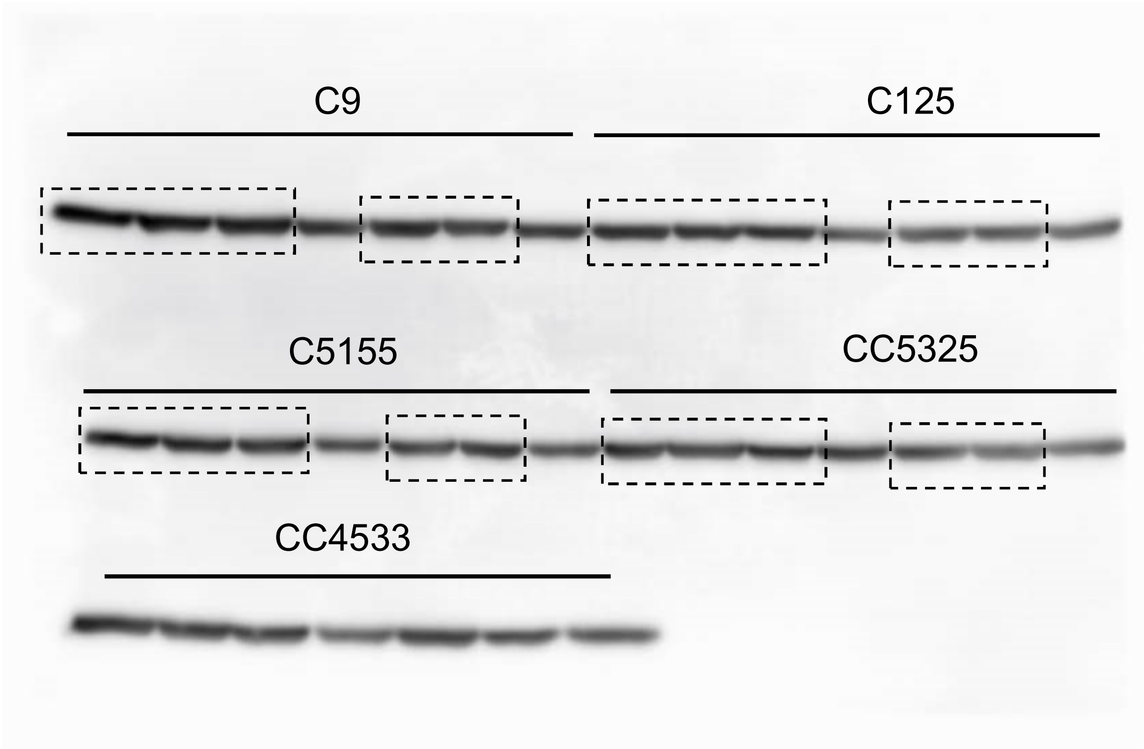

Figure S4

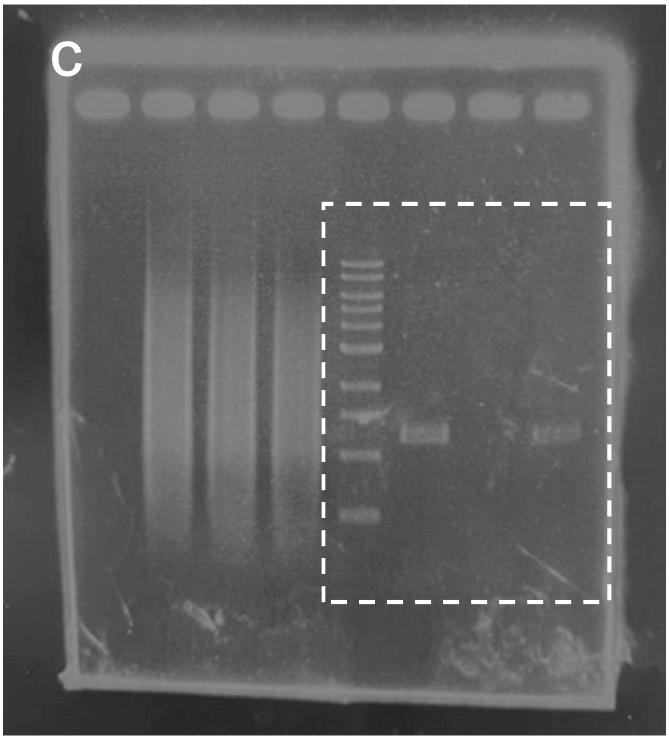

A larger image of the agarose gel in Fig. S4b is not available.
